# Genome-Wide Identification, Expression Analysis, and Potential Roles under Abiotic Stress of the *YUCCA* Gene Family in Mungbean (*Vigna radiata* L.)

**DOI:** 10.3390/ijms24021603

**Published:** 2023-01-13

**Authors:** Ranran Wu, Jingbin Chen, Yun Lin, Qiyuan Jia, Yingjian Guo, Jinyang Liu, Qiang Yan, Chenchen Xue, Xin Chen, Xingxing Yuan

**Affiliations:** 1 Institute of Industrial Crops, Jiangsu Academy of Agricultural Sciences, Nanjing 210014, China; 2 Jiangsu Key Laboratory for Horticultural Crop Genetic Improvement, Nanjing 210014, China; 3 College of Life Sciences, Nanjing Agricultural University, Nanjing 210095, China

**Keywords:** mungbean, *YUCCA* gene family, identification, expression profiles, abiotic stress, transgene

## Abstract

*YUCCA*, belonging to the class B flavin-dependent monooxygenases, catalyzes the rate-limiting step for endogenous auxin synthesis and is implicated in plant-growth regulation and stress response. Systematic analysis of the *YUCCA* gene family and its stress response benefits the dissection of regulation mechanisms and breeding applications. In this study, 12 *YUCCA* genes were identified from the mungbean (*Vigna radiata* L.) genome and were named based on their similarity to *AtYUCCAs*. Phylogenetic analysis revealed that the 12 *VrYUCCAs* could be divided into 4 subfamilies. The evidence from enzymatic assays in vitro and transgenetic *Arabidopsis* in vivo indicated that all the isolated *VrYUCCAs* had biological activity in response to IAA synthesis. Expression pattern analysis showed that functional redundancy and divergence existed in the *VrYUCCA* gene family. Four *VrYUCCAs* were expressed in most tissues, and five *VrYUCCAs* were specifically highly expressed in the floral organs. The response toward five stresses, namely, auxin (indole-3-acetic acid, IAA), salinity, drought, high temperatures, and cold, was also investigated here. Five *VrYUCCAs* responded to IAA in the root, while only *VrYUCCA8a* was induced in the leaf. *VrYUCCA2a*, *VrYUCCA6a*, *VrYUCCA8a*, *VrYUCCA8b*, and *VrYUCCA10* seemed to dominate under abiotic stresses, due to their sensitivity to the other four treatments. However, the response modes of the *VrYUCCAs* varied, indicating that they may regulate different stresses in distinct ways to finely adjust IAA content. The comprehensive analysis of the *VrYUCCAs* in this study lays a solid foundation for further investigation of *VrYUCCA* genes’ mechanisms and applications in breeding.

## 1. Introduction

Auxin/indole-3-acetic acid (IAA) is one of the major endogenous phytohormones, playing a critical role in regulating plant growth and development as well as the stress response [1], and it results from the coordinated actions of auxin biosynthesis, metabolism, transport, and signaling [2]. During the plant life cycle, IAA adjusts almost all aspects of biological processes, including embryogenesis [3], shoot and root formation [4], leaf development [5], floral development and fertility [6,7], and fruit initiation [8]. Additionally, auxin homeostasis is essential for plants to adapt to biotic and abiotic stresses, such as pathogens, herbivory, temperature, salt, and drought [1,9,10].

In plants, both tryptophan (Trp)-dependent and -independent pathways contribute to the auxin de novo synthesis, and the former is very well understood [11]. There are four distinct routes that involve different metabolic intermediates, namely, tryptamine (TAM), indole-3-pyruvic acid (IPA), indole-3-acetaldoxime (IAOx), and indole-3-acetamide (IAM) [12]. The IPA pathway (two steps) is mainly responsible for IAA biosynthesis in plants, in which Trp is first converted into IPA by tryptophan aminotransferase (TAA), and then YUCCA catalyzes the rate-limiting irreversible reaction, producing IAA from IPA [11,13].

YUCCA belongs to class B flavin-dependent monooxygenases (FMOs; EC 1.14.13.8), the only class of FMOs found in plants based on the current status of available sequencing data [12]. It has been demonstrated that YUCCA proteins share several conserved motifs, including the FAD-binding motif (GXGXXG), FMO-identifying sequence (FXGXXXHXXXY/F), NADPH-binding motif (GXGXXG), ATG-containing motif1 (Y_(X)7_ATGEN_(X)5_P), and ATG-containing motif2 (DxxxxATGY) [12,14]. Among these motifs, the FAD- and NADPH-binding motifs are central to YUCCA activity and have the same characteristic structure in amino acid sequences [11]. The FMO-identifying sequence, a key sequence in all known plant FMOs, contributes to NADPH binding [15].

To date, genome-wide identification of *YUCCA* family genes has been performed in more than 30 plants species, such as *Arabidopis thaliana* [16], *Oryza sativa* [17], *Zea mays* [18], *Cucumis sativus* [2], *Glycine max* [19], *medicago truncatula* [20], *Populus trichocarpa* [9], *Lilium* [21], *malus domestica* [22], *Triticum aestivuml* [14], *Gossypium* spp. [23], and *Brassica napus* [24]. However, knowledge about the *YUCCA* gene family and its potential roles in mungbean is limited. The biological functions of *YUCCA* genes have been demonstrated thoroughly in some species, especially in model plants. In *Arabidopsis*, the *YUCCA*(*YUC*) gene family includes 11 members with overlapping functions in plant development. In 2001, *AtYUC1* was first identified from a dominant *Arabidopsis* mutant *yucca* involved in the production of IAA [25]. Subsequent functional studies on the loss-of-function or gain-of-function of *YUCCA* genes in *Arabidopsis* indicated that they played a vital role in developmental processes, including floral patterning and vascular formation (e.g., *AtYUC1,2,4,6*) [16], embryogenesis and leaf formation (e.g., *AtYUC1,4,10,11*) [26], hypocotyl elongation (e.g., *AtYUC5*) [27], and the regeneration of root systems (e.g., *AtYUC9*) [28]. *AtYUC6* and *AtYUC7* were reported to confer drought tolerance depending on distinct mechanisms [10,29]. AtYUC6 played a dual role in regulating plant growth as an auxin biosynthetic enzyme and conveying drought response as a thiol-reductase (TR)-like protein, which functioned through ROS regulation [29]. While root *AtYUC7* could be induced by drought in an abscisic acid (ABA)-dependent manner, the auxin level elevation promoted lateral root growth to protect plants from drought [10]. It was reported that *AtYUC8* and *AtYUC9* were upregulated under high temperatures [1,30], and constitutively overexpressing these two genes showed less damage after spider mite infestation [31]. Under high temperatures, the transcriptional regulator phytochrome-interacting factor 4 (*PIF4*) directly induced *AtYUC8* expression to elevate endogenous free IAA levels, and the plant exhibited hypocotyl elongation [30]. In rice, 14 *YUCCA* genes have been identified, with a crucial role in crown root development (e.g., *OsYUCCA1*) [32], leaf morphogenesis (e.g., *OsNAL7*/*OsYUCCA8*) [33], maintaining water homeostasis and appropriate root-to-shoot ratios (e.g., *OsCOW1*) [34], and auxin-mediated anther dehiscence (e.g., *OsYUCCA4*) [35]. Two research teams have reported that 22 *YUCCA* genes were isolated from soybean, though with different naming rules [19,20]. There were, therefore, four controversial sequences. *GmYUCCA2* (*Glyma.03G208900*) and *GmYUCCA21* (*Glyma.19G206200*), identified in 2017 [19], were not included in the 2019 research, replaced by *GmYUC5-8-9a* (*Glyma.10G041800*) and *GmYUC5-8-9b* (*Glyma.13G128800*) [20]. Therefore, the soybean genome contains at least 24 members of the *YUCCA* gene family. Overexpression of *GmYUCCA5* (*Glyma.04G070100*) in *Arabidopsis* displayed downward curling of the leaf-blade margin, evident apical dominance, higher plant height, and shorter length of siliques [19]. It was also proven that *GmYUC2a* (*Glyma.08g038600*) acted as a vital part in regulating both root growth and nodulation by modulating the auxin balance in soybean [20].

Mungbean is an important warm-season grain legume with rich nutrition and short duration, facilitating environmental sustainability [36]. Our previous study showed that a *YUC4*-like protein regulated the chasmogamous flower trait in mungbean [37], indicating the crucial roles of *YUCCA* genes in developmental regulation. In this study, we systematically identified 12 *YUCCA* family genes (named as *VrYUCCA*) in the mungbean genome and investigated their gene structures, expression patterns, biological activity in vitro and in vivo, and potential roles under abiotic stresses. This research can provide valuable information about the functional explanation of the *YUCCA* gene family in mungbean, contributing to the application of these genes in mungbean genetic improvement.

## 2. Results

### 2.1. Identification of the YUCCA Gene Family in Mungbean and Structure Analysis

Based on the output of the Gcorn plant, BLAST homology search, and conserved domain analysis, 12 *VrYUCCA* genes were isolated from the mungbean genome (Table S1), named according to their similarity to *Arabidopsis YUCCA* genes [16]. No homologs for *AtYUC1*, *AtYUC5*, *AtYUC7*, or *AtYUC9* were found in mungbean. Meanwhile, there were three homologs for *AtYUC8* and two each for *AtYUC2*, *AtYUC4*, and *AtYUC6*. The deduced amino acid lengths of the 12 VrYUCCA proteins ranged between 385 and 440 aa, with the pI between 7.96 and 9.54 and the molecular weight between 43.14 and 49.89 kD.

**Figure 1:**
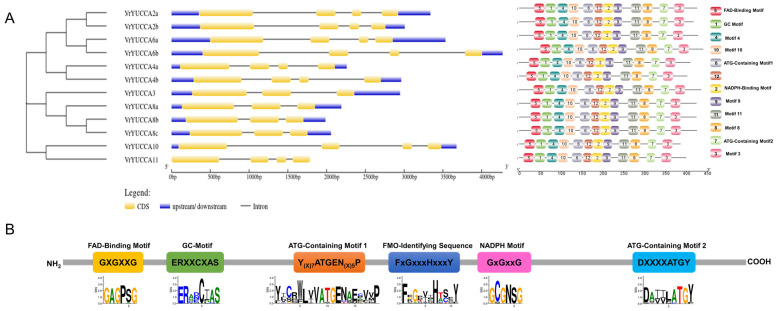
The gene structure and conserved motifs of the *YUCCA* gene family in mungbean. (**A**) Phylogenetic tree (**left**), gene structure (**middle**), and conserved motifs (**right**) of 12 *VrYUCCAs*. (**B**) The schematic diagram of six conserved protein motifs.

To investigate the diversity of the gene structures of the 12 *VrYUCCAs*, the exon and intron configurations were determined through visualized analysis using the GSDS tool. *VrYUCCA3*, *VrYUCCA8a*, *VrYUCCA8b,* and *VrYUCCA8c*, which were clustered in the same group, each contained three exons and two introns. The other *VrYUCCAs* all had four exons and three introns (Figure 1A). The conserved protein motifs were analyzed by MEME, and a total of 12 motifs are exhibited here (Figure 1A). All six of the recognized conserved motifs, including the FAD-binding motif, GC motif, ATG-containing motif1, FMO-identifying sequence, NADPH-binding motif, and ATG-containing motif2, are shown in a both schematic diagram and sequence alignment (Figure 1B and Figure S1).

### 2.2. Phylogenetic Analysis of YUCCAs from Multiple Species

**Figure 2:**
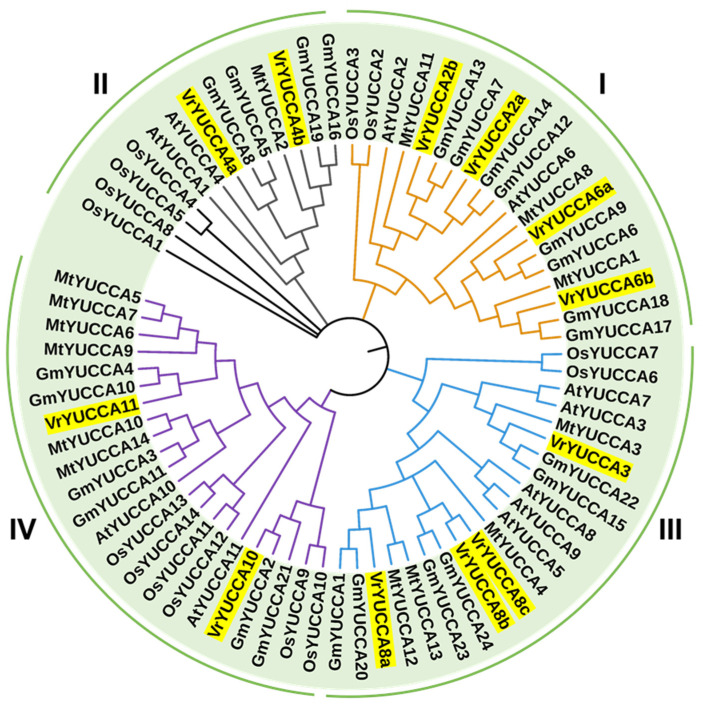
Phylogenetic tree of 75 YUCCA proteins from mungbean, *Arabidopsis*, soybean, rice, and Medicago. Vr, *Vigna radiata*; At, *Arabidopsis thaliana*; Gm, *Glycine max*; Os, *Oryza sativa*; Mt, *Medicago truncatula*. Each color represents a branch, and 12 VrYUCCAs are highlighted.

To further describe the evolutionary relationship, we performed an unrooted phylogenetic tree of YUCCA proteins from mungbean, *Arabidopsis*, soybean, rice, and Medicago (Figure 2). A total of 75 YUCCA proteins from five plant species were classified into four subfamilies (I to IV), which was consistent with previous research on *Arabidopsis* [16] and soybean [19]. VrYUCCA2a, VrYUCCA2b, VrYUCCA6a, and VrYUCCA6b were grouped together in subfamily I; VrYUCCA4a and VrYUCCA4b were placed in subfamily II; VrYUCCA3, VrYUCCA8a, VrYUCCA8b, and VrYUCCA8c were located in subfamily III; and VrYUCCA10 and VrYUCCA11 were placed in subfamily IV. At the same time, four rice YUCCA proteins (OsYUCCA1, OsYUCCA4, OsYUCCA5, and OsYUCCA8) did not cluster well with the YUCCA proteins in subfamily II. Therefore, these four proteins could also be classified into another new subfamily, subfamily V. Among these five analyzed plant species, the evolutionary relationship of VrYUCCA proteins was closest to GmYUCCAs. The sequences information of all the YUCCAs is listed in Supplementary Data S1.

### 2.3. Expression Pattern of VrYUCCAs in Mungbean

**Figure 3:**
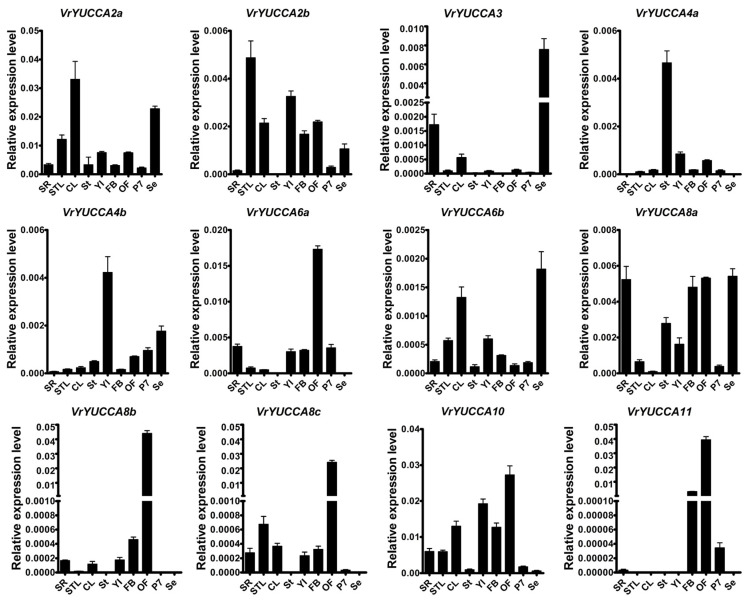
Expression pattern of *VrYUCCAs* in different tissues. SR, seeding root; STL, seedling true leaf; CL, compound leaf; St, stem; YI, young inflorescence; FB, flower bud; OF, opening flower; P7, 7-day pod; Se, seed. The *VrACTIN3* gene (*Vradi03g00210*) was used as the internal control to normalize the real-time PCR data. The 2^−ΔCT^ method was used to analyze relative expression level. Error bars indicated SDs (standard deviations) from three biological repetitions.

To explore the potential biological functions of *VrYUCCA* genes during mungbean development, we analyzed their expression patterns in different tissues, including seedling root (SR), seedling true leaf (STL), compound leaf (CL), stem (St), young inflorescence (YI), flower bud (FB), opening flower (OF), 7-day pod (P7), and seed (Se), using real-time PCR (Figure 3). Several *VrYUCCAs* were constitutively expressed in all tissues examined, including *VrYUCCA2a*, *VrYUCCA6b*, *VrYUCCA8a,* and *VrYUCCA10*. Most *YUCCAs* had an extremely low expression in the stems except for *VrYUCCA2a*, *VrYUCCA4a,* and *VrYUCCA8a*; among them, *VrYUCCA4a* was specifically expressed. *VrYUCCA3* was preferentially expressed in seed. There were five *VrYUCCAs* specifically enriched in flower organs, including *VrYUCCA4b* (IY), *VrYUCCA6a* (OF), *VrYUCCA8b* (OF), *VrYUCCA8c* (OF), and *VrYUCCA11* (FB and OF). These results suggest that functional redundancy and divergence exist in the *VrYUCCA* gene family, and during different stages of growth or organs, distinct *VrYUCCAs* might play a major role.

### 2.4. VrYUCCA Proteins Showed Biological Activity Both In Vitro and In Vivo

**Figure 4:**
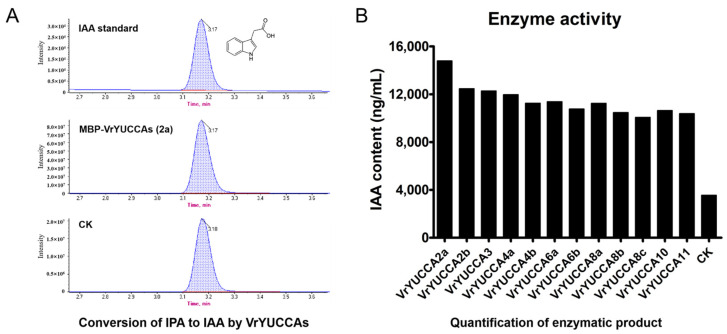
Enzymatic assay of VrYUCCAs with the substrate IPA analyzed by ESI-HPLC-MS/MS. (**A**) The HPLC profile for IAA standard and the enzymatic products. The profile of VrYUCCA2a reaction was selected as a representative here, and the results of other VrYUCCAs are shown in Figure S2. CK: the enzyme reaction mixture containing a boiled protein. The relatively lower HPLC profile in CK was due to spontaneous conversion of IPA to IAA at room temperature. (**B**) The statistical results of the IAA content in enzymatic reaction mixture of VrYUCCAs.

**Figure 5:**
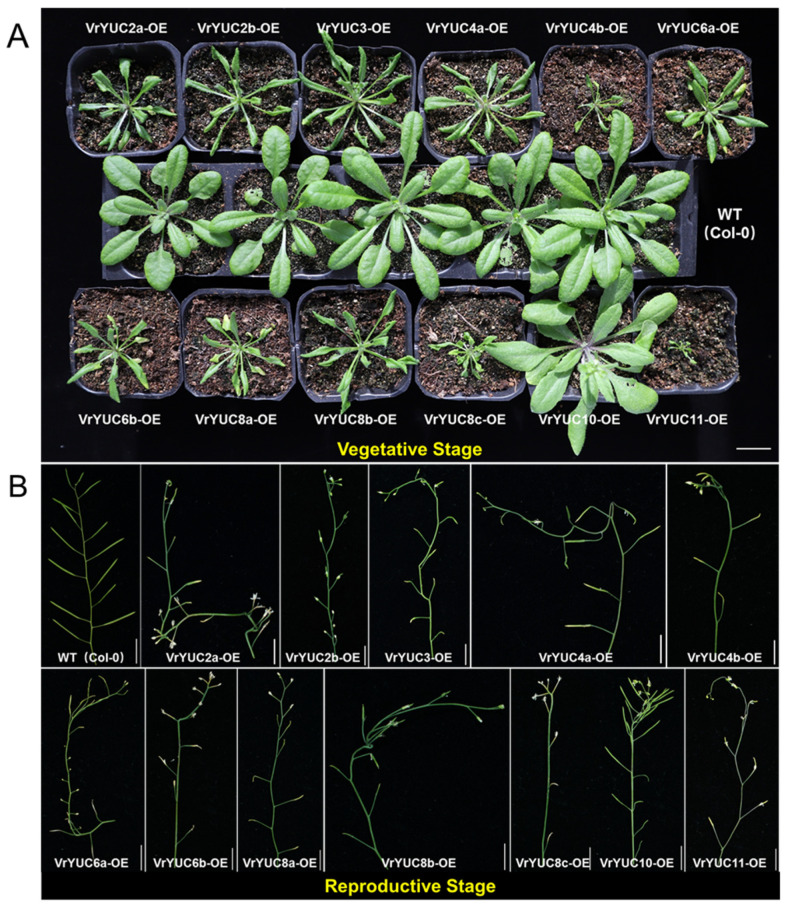
The phenotypic analysis of 35S::*VrYUCCAs* transgentic *Arabidopsis* during vegetative period (**A**) and reproductive growth stage (**B**). OE: overexpression line. Bar = 1 cm.

It was reported that YUCCA proteins might function in two possible routes to produce IAA, with IPA or TAM as the substrates, respectively [13,25]. To investigate the catalytic substrate of VrYUCCA proteins, enzyme activity detection was conducted in vitro. The results indicated that all 12 VrYUCCAs could catalyze IPA to produce IAA (Figure 4 and Figure S2), but they showed no activity toward TAM (Figure S3). Thus, in mungbean, VrYUCCAs participated in the IPA pathway to synthesize IAA. Further, to examine their biological activity in vivo, *VrYUCCAs* were overexpressed in *Arabidopsis* (Col-0, wild type; WT) and driven by the 35S strong promoter. The T_1_ generation of most *VrYUCCAs* transgenic plants showed typical auxin-related phenotypes (Figure 5), such as narrow rosette leaves, downward curling of the leaf-blade margin, twisted stem, and shorter or no siliques. What was noteworthy was that 35S::*VrYUCCA10*-transgenic plants showed a distinct phenotype, as their leaves were broader and slightly curled, with denser siliques on the top of the stem. These results indicated that all 12 of the isolated *VrYUCCA* genes had a veritable role in IAA synthesis.

### 2.5. VrYUCCAs Expression in Response to Abiotic Stress Treatments

**Figure 6:**
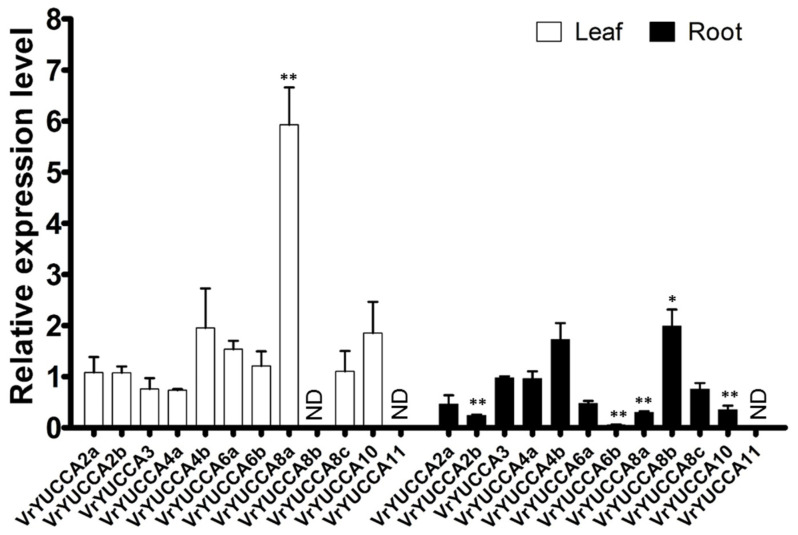
Effect of IAA on the expression of *VrYUCCAs*. The asterisks indicate statistical significance (*, *p* < 0.05; **, *p* < 0.01) compared with each CK (standardized as 1). Error bars indicate SDs from 3 biological repetitions. ND: not detectable.

To investigate the response of *VrYUCCAs* toward surroundings stresses, 12-day-old mungbean seedlings were exposed to IAA (1 µM), high salinity (200 mM NaCl), drought (15% PEG6000), heat (35 °C), and chilling (4 °C) for some time. The expression of *VrYUCCA11* could not be detected well in the root and leaf, even under these stresses, due to its specific expression in flower organ. As shown in Figure 6, *VrYUCCA2b, VrYUCCA6b*, *VrYUCCA8a*, and *VrYUCCA10* were inhibited markedly in the root by IAA, while *VrYUCCA8b* was induced. In the leaf, only the expression of *VrYUCCA8a* showed a highly significant increase.

**Figure 7:**
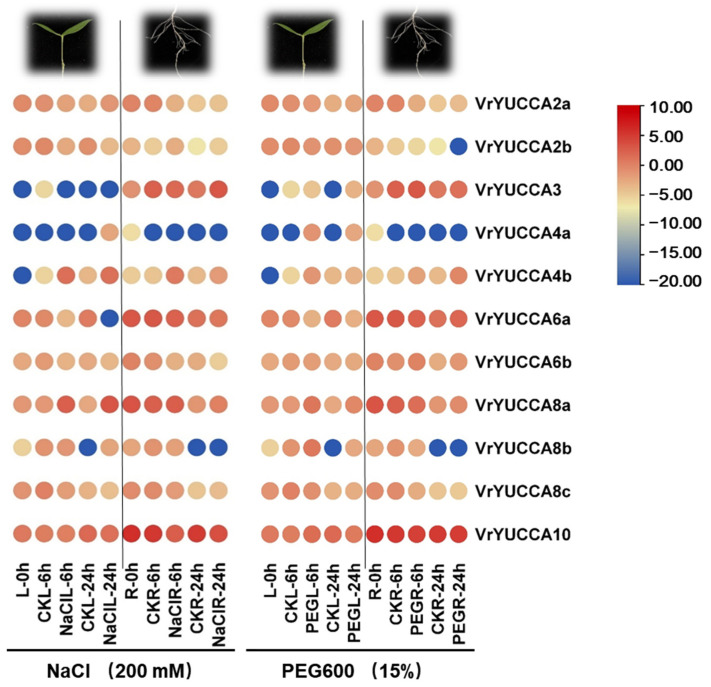
*VrYUCCA* genes expression during salt and drought stress. Values (RNA-seq data), in fragments per kilobase of transcript per million reads mapped (FPKM), used logarithm (LOG 2) for the heat map. L: leaf; R: root.

**Figure 8:**
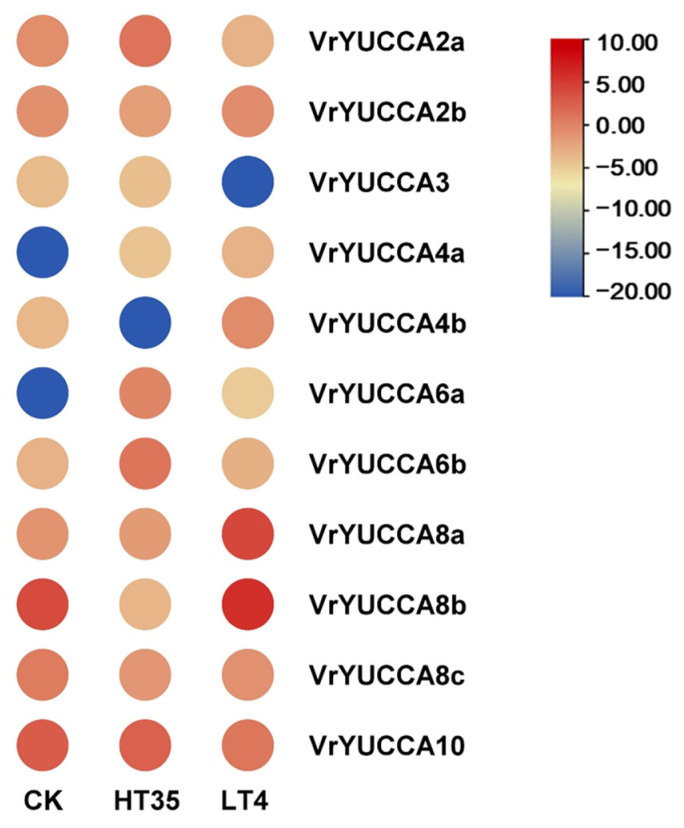
*VrYUCCA* genes expression during high and low temperatures. Values (RNA-seq data), in fragments per kilobase of transcript per million reads mapped (FPKM), used logarithm (LOG 2) for the heat map. CK: seedlings grown under 25 °C; HT35: seedlings grown under high temperatures, 35 °C; LT4, seedlings grown under low temperatures, 4 °C.

As for the other abiotic stresses, all the analyzed *VrYUCCA* genes showed a visible increase and decrease under high-salinity treatment (Figure 7 and Figure S4). Both at 6 h and 24 h after NaCl treatment, *VrYUCCA4b* and *VrYUCCA8a* had high expression in the leaf; meanwhile, the expression of *VrYUCCA2b* and *VrYUCCA6a* was inhibited significantly, indicating their main roles under salt stress. Several genes seemed to only function under long-term stress, such as *VrYUCCA4a* and *VrYUCCA8b* in the leaf. Additionally, *VrYUCCA2a*, *VrYUCCA6b*, *VrYUCCA8c*, and *VrYUCCA10* mainly responded to salinity in the root. Under drought stress (Figure 7 and Figure S5), *VrYUCCA6a* was inhibited, while *VrYUCCA8a* and *VrYUCCA8b* were induced in the leaf. In the root, however, *VrYUCCA2a*, *VrYUCCA8a*, *VrYUCCA8c*, and *VrYUCCA10* might play a crucial part. When the mungbean seedlings were exposed to high temperatures, the expression of *VrYUCCA2a*, *VrYUCCA6a,* and *VrYUCCA6b* markedly increased, while that of *VrYUCCA8b* and *VrYUCCA8c* was inhibited. Cold prominently induced the expression of *VrYUCCA8a* and *VrYUCCA8b*. Meanwhile the expression of *VrYUCCA2a* and *VrYUCCA10* declined (Figure 8 and Figure S6). Overall, *VrYUCCA2a*, *VrYUCCA6a*, *VrYUCCA8a*, *VrYUCCA8b,* and *VrYUCCA10* proved to be sensitive to all four treatments and might play a major role under abiotic stresses.

## 3. Discussion

YUCCA, a key rate-limiting enzyme for IAA synthesis, has been widely characterized in quite a few higher plants [11], existing in forms of families in different organisms, with numbers ranging from a few (e.g., 6 in tomato [38]) to dozens (e.g., 57 in *Brassica napus* [24] and 63 in wheat [14]), except for petunia, which only has one *YUCCA* gene in its genome [39]. Reports about *YUCCA* genes in mungbean are limited. Previously, a YUC4-like protein (*Vradi06g12650*) was reported to be responsible for the mungbean chasmogamous mutant (CM) [37], indicating the vital role of *VrYUCCAs* during plant growth and development. In this study, we identified 12 *VrYUCCAs* in the mungbean genome, named based on their similarity to *AtYUCCAs*. Homologous genes of *AtYUC1, AtYUC5, AtYUC7,* and *AtYUC9* were absent, but two each for *AtYUC2*, *AtYUC4,* and *AtYUC6* and three for *AtYUC8* were isolated in mungbean (Figure 1). This phenomenon also exists in apple and cucumber [2,22], which seems normal for gene family studies, and chromosomal fragment deletion and genetic duplication might be the causative factors of this phenomenon. Further, multiple copies of the *VrYUCCAs* displayed distinct expression patterns (Figure 3), indicating their functional differentiation during evolution.

The 12 VrYUCCA proteins could be divided into four groups (Figure 2), possessing the conserved motifs of the *YUCCA* gene family. Subfamily I (*YUC2*–*YUC6* group) and subfamily II (*YUC1*-*YUC4* group) were reported to function in the inflorescence apex and flower in *Arabidopsis*, and *AtYUC4* was specifically expressed in the floral meristems [16]. We previously obtained one mungbean mutant related to *VrYUCCA4b* that had developmental defects in its flower organs [37]. *VrYUCCA4b* (*Vradi06g12650*) seems to have a similar role in floral development, which is also highly expressed in young inflorescence (Figure 3). In *Arabidopsis*, the *Atyuc4* single mutant displayed the normal phenotype, but *Atyuc1yuc4* showed severe defects in its floral organs and fertility [16]; in mungbean, the homologous genes of *AtYUC1* were vacant, and other *VrYUCCAs* including *VrYUCCA4a* seemed not to complement the function of *VrYUCCA4b*, resulting in the petal-loss trait of CM (the *Vryucca4b* mutant) [37]. Thus, *YUCCA* paralogous genes have functional redundancy to ensure normal plant development. Additionally, in *Isatis indigotica* Fort, *IiYUCCA1*, and *IiYUCCA4* were also mainly detected in reproductive organs [40]. In *Brassica napus*, *BnYUC4* was also highly expressed in early flower buds [24]. Thus, the expression pattern and function of YUCCAs seem to be conserved. It was reported that subfamily III (*YUC3-7-8-5-9* group) showed distinct expression patterns during root development in *Arabidopsis* [41,42]. Interestingly, in the root defects of these five *YUCs* (*YUC3-7-8-5-9*), a multiple mutant (*yucQ)* could be rescued by expressing a *YUCCA* gene in subfamily III rather than in other subfamily members [41]. At the same time, in mungbean, a high transcript of *VrYUCCA3* was detected in its seeds (Figure 3), implying a likely vital role during seed formation. *VrYUCCA8b* and *VrYUCCA8c* seemed like gene clusters located adjacently on chromosome 8, and both were expressed extremely highly during flower opening. However, *VrYUCCA8a* had a relatively high expression in the root. *VrYUCCA10* and *VrYUCCA11* were found in subfamily IV (*YUC10-11* group), which was assumed to be the most conserved group of *YUCCAs* in the plant kingdom [42]. *AtYUC10* and *AtYUC11* mainly expressed in siliques and apices and were suggested to have overlapping functions with *AtYUC1* and *AtYUC4* during embryogenesis [26]. In mungbean, the relative transcript level of *VrYUCCA10* was the highest in all tissues (Figure 3), hinting at its essential role during the whole life cycle. At the same time, the transcript of *VrYUCCA11* was examined only in the floral organs and pods (Figure 3). In plants, *YUCCA11* seemed to be specifically involved in the floral organs, embryogenesis, and fruit development. In cucumber, *CsYUC11* was specifically enriched in the opened male flower (mainly detected in the tapetum cell layer and the microspores in the anthers), and transgenic *Arabidopsis* lines of 35S::*CsYCU11* displayed defective pollens and reduced fertility [2]. In peach, the transcript of *PpYUC11* increased during the late ripening stage in melting flesh peaches, implying its vital function during fruit ripening [43]. The number of *YUCCA11* homologous genes increased in several *Rosaceae* species, such as apple (six *YUCCA11*) and white pear (seven *YUCCA11*). *MdYUCCA11a*, *MdYUCCA11b,* and *MdYUCCA11d* showed high expression in young fruit, the receptacle, and the pistil, respectively [22]. Thus, like *YUCCA* in other species, distinct *VrYUCCA* participated in specific biological processes with redundancy and differential functions.

In this study, we verified 12 VrYUCCA proteins’ enzyme activity both in vivo and in vitro. The substrate of YUCCA was controversial. Initially, it was demonstrated that the *Arabidopsis* YUCCA1 protein could catalyze the *N*-hydroxylation of TAM to form *N*-hydroxy-TAM in vitro [25]. Meanwhile, later in vivo research showed that YUCCA1 also catalyzed the oxidative decarboxylation of IPA to form IAA [44,45]. Subsequently, a growing number of studies have suggested that the real substrate of YUCCA was IPA rather than TAM [13,46,47]. Here, VrYUCCA exhibited explicit activity toward IPA, but no novel compound was produced toward TAM in vitro (Figure 4 and Figure S3). The 35S::*VrYUCCAs* transgenetic *Arabidopsis* lines all showed typical IAA over-accumulation defects (Figure 5), indicating the authentic function of the *VrYUCCAs* for IAA synthesis in vivo. No seeds were harvested from the 35S::*VrYUCCA11* line, which displayed the most severe abnormity during T_1_ generation. Notable was, the 35S::*VrYUCCA10* line displayed less of an effect in the vegetative growth stage (even stronger than WT), but the siliques showed apex density. Whether this line has special potential advantages during developmental processes or under stress need to be studied further.

Auxin, a central regulator involved in almost all aspects of the plant life cycle, helps plants to adaptive external stresses [1,48]. YUCCA protein catalyzes IPA to produce IAA, and IAA can also feedback modulate *YUCCA* expression. Here, five *VrYUCCA* genes responded to IAA stimulation, which mainly functions in the root. Notably, only *VrYUCCA8a* was induced in the leaf, but it was significantly inhibited in the root (Figure 6), indicating that it might control gene expression by regulating hormone signaling through complex crosstalk, as reported in soybean [19]. In apple and soybean, the homologous gene of *VrYUCCA8a* also displayed sensitivity to hormone treatments [19,22], indicating its functional conservation in the plant kingdom. Additionally, *YUCCA* was also proven to be involved in IAA-mediated stress response [2,9,31]. In this study, we investigated the potential roles of *VrYUCCAs* in stress response. More *VrYUCCAs* were sensitive under salt stress than drought treatment (Figure 7, Figures S5 and S6). *VrYUCCAs* showed different expression patterns to respond to the same stress; for example, leaf *VrYUCCA2b* and *VrYUCCA6a* were inhibited markedly under high salinity, while the expression of *VrYUCCA4b* and *VrYUCCA8a* increased significantly. These results hinted that the regulation mechanism of the stress response by YUCCA-mediated IAA synthesis is complex and can even antagonize to create a buffering system for endogenous auxin synthesis, as reported in cucumber [2]. Regarding heat and chilling, *VrYUCCA2a*, *VrYUCCA6a, VrYUCCA6b, VrYUCCA8a,* and *VrYUCCA8b* might act as the main regulators. In *Arabidopsis* and apple, *PIF4* could specifically bind to the G-box motif (CACGTG) in the promoter of *YUCCA8* to activate its expression, which was deemed a conserved molecular mechanism [22,30]. In cucumber, *CsYUC8* and *CsYUC9* were selectively upregulated after being exposed to 38 °C for 3 h [2]. Remarkably, *VrYUCCA8a* and *VrYUCCA8b* were markedly induced by low temperatures rather than heat, similar to that in *Populus* [9], suggesting that high-temperature regulatory mechanisms might be diverse in mungbean. It is possible that several *VrYUCCAs* have a dual role, like *AtYUC6* [29], and transmit stress signals through another functional domain mediated by ROS or hormone crosstalk, such as ABA and SA, which needs further exploration.

Overall, the *YUCCA* gene family is relatively conservative among plants, and the homologous genes in different species tend to act similarly in their roles during plant growth and stress responses. The crucial role of *YUCCAs* in the auxin pathway is beyond doubt, so further in-depth research should be conducted to investigate its functional mechanism and application value.

## 4. Materials and Methods

### 4.1. Identification, Structure, and Motif Analysis of VrYUCCA Genes

Multiple methods were used to identify mungbean YUCCAs. Firstly, Gcorn plant (http://www.plant.osakafu-u.ac.jp/~kagiana/gcorn/p/index18.html) (accessed on 1 September 2020) [49], a database of plant gene phylogeny, was used to search *YUCCA* information in mungbean genome. Second, the amino acid sequences of the 11 *Arabidopsis* YUCCA (AtYUCs) were downloaded from TAIR (https://www.arabidopsis.org/index.jsp) (accessed on 1 September 2020) and then used as BlastP queries against mungbean genome database (http://plantgenomics.snu.ac.kr/mediawiki-1.21.3/index.php/Main_Page) (accessed on 1 September 2020) to reconfirm the *VrYUCCAs*. Then, the online tool SMART (http://smart.embl-heidelberg.de/) (accessed on 1 October 2020) was used to confirm whether the predicted VrYUCCA proteins had the FMO-like domain. Finally, the sequences information of *VrYUCCAs* was downloaded from NCBI (https://www.ncbi.nlm.nih.gov) (accessed on 1 October 2020). The online tool GSDS (http://gsds.gao-lab.org/) (accessed on 1 October 2020) was used to determine the gene structure. The online MEME suite (https://meme-suite.org/) (accessed on 1 October 2020) was used to identify the conserved motifs shared among VrYUCCA proteins, which were then visualized with TBtools, and the repeats logos were obtained through WEBLoGo (http://weblogo.threeplusone.com/create.cgi) (accessed on 1 October 2020). The theoretical pI (isoelectric point) and Mw (molecular weight) of VrYUCCAs were predicted using ExPASY (https://www.expasy.org/) (accessed on 1 October 2020). BaCelLo (http://gpcr.bio-comp.unibo.it/bacello/index.htm) (accessed on 1 October 2020) was used to predict the subcellular localization. Multiple sequence alignment of the identified VrYUCCA sequences was performed using MEGA-X and ClustalX2 and then visualized using ESPript 3.0 (https://espript.ibcp.fr/ESPript/cgi-bin/ESPript.cgi) (accessed on 1 October 2020). 

### 4.2. Phylogenetic Analysis of YUCCA Genes from Multiple Species

The amino acid sequences of YUCCA from *Arabidopsis* (11), *Oryza sativa* (14), *Glycine max* (24), and *Medicago truncatula* (14) were downloaded from TAIR, RAP-DB (The Rice Annotation Project Database), NCBI, and phytozome (https://phytozome-next.jgi.doe.gov/) (accessed on 1 November 2020), respectively. The phylogenetic tree was constructed using MEGAX software with the neighbor-joining (NJ) method with 1000 bootstrap replicates. Additionally, it was optimized on the website iTOL (https://itol.embl.de/) (accessed on 1 November 2020).

### 4.3. Plant Materials and Treatment

The seedlings of mungbean cultivar ‘Sulyu 1’ were grown in the illumination incubator at 25 °C with 16 h light/8 h dark photoperiod. Adult mungbean was planted in experimental plots at Jiangsu Academy of Agricultural Sciences, Nanjing, China (32°21′ N, 118°53′ E).

Seedling root, seedling true leaf, stem, compound leaf, young inflorescence, flower bud, opening flower, pod (7 d), and dry seed were sampled from mungbean seedlings (12 d) or adult plants. All the tissues were immediately frozen in liquid nitrogen, and each sample was collected independently three times for further *VrYUCCA* expression pattern analysis using real-time PCR.

For IAA treatment, 12-day-old mungbean seedlings were soaked in liquid MS (Murashige and Skoog) medium with 1 μM IAA and liquid MS medium only (as the control) for 4 h. The treated roots and leaves were sampled separately after flushing with ddH2O and immediate freezing in liquid nitrogen. Treatment was repeated three times. The expression of *VrYUCCA* genes was then examined by real-time PCR.

For high-salinity and drought treatments, 12-day-old mungbean seedlings were soaked in liquid MS medium with 200 mM NaCl and 15% (*w*/*v*) polyethylene glycol (PEG) 6000 for 6 h and 24 h, respectively. The seedlings soaked in liquid MS medium served as the control, and the treated roots and leaves were sampled separately after flushing with ddH2O and immediate freezing in liquid nitrogen. Treatment was repeated three times. Then, the expression of *VrYUCCA* genes was analyzed using RNA-seq (BGI, Shenzhen, China).

For high- and low-temperature treatments, 12-day-old mungbean seedlings were placed in an incubator at 35 °C and 4 °C, respectively, for 24 h. The seedlings grown under 25 °C were taken as a control. Additionally, the treated leaves were sampled and immediately frozen in liquid nitrogen. The expression of *VrYUCCA* genes was detected using RNA-seq (BGI, Shenzhen, China).

### 4.4. Expression Analysis Using Real-Time PCR

Total RNA was extracted using Polysaccharide polyphenol Plant total RNA Extraction Kit (PD Biotech, Shanghai, China), and cDNAs were synthesized using Goldenstar^®^ RT6 cDNA Synthesis Kit Ver.2 (TSINGKE, Beijing, China). Real-time PCR was performed using ChamQ SYBR qPCR Master Mix (Vazyme, Nanjing, China) on an ABI prism 7500 real-time PCR System. Mungbean *VrACTIN3* (*Vradi03g00210*) and *Arabidopsis ACTIN2* (*AT3G18780*) were selected as reference genes to normalize the expression data. The 2^−ΔCT^ or 2^−ΔΔCT^ methods were used to analyze relative expression levels. The results were calculated from three biological replicates and three technical replicates. Statistical analysis and plot drawing were conducted using Excel and GraphPad Prism 5 software. Heat maps were drawing by TBtools after the FPKM value taken logarithm (LOG 2). The primers used in this study are listed in Table S2.

### 4.5. Prokaryotic Expression, Purification, and Enzymatic Assays of VrYUCCA

The full-length CDS sequence of each *VrYUCCA* gene was ligated into a pMAL-c2x expression vector (NEB, MBP-tag). Then, the recombinant VrYUCCA proteins were expressed in *E. coli* (BL21, DE3) and purified by dextrin Sepharose affinity chromatography (Amylose resin, NEB), in accordance with the instructions described previously [50]. The protein concentration was determined using the Bradford method with BSA as the standard.

The enzymatic assays were conducted by referring to a previous study [47]. A total 40 µL reaction system containing 1~2 µg VrYUCCA protein, 50 mM Tris-HCl (pH 7.4), 10 mM NADPH/NADP, and 2 mM of the substrates IPA or TAM was incubated at 30 °C for 4 h. A mixture containing one boiled VrYUCCA protein was served as the control. The products were analyzed on silica-gel-coated TLC plates with chloroform/methanol/acetic acid (75:20:5) as the mobile phase. The TLC plates were stained using Ehrlich’s reagent (Sigma, 02560-500ML, St. Louis, MO, USA). Meanwhile, the products with IPA as the substrate were also detected by ESI-HPLC-MS/MS conducted by Nanjing WEBiolotech Biotechnology Co., Ltd. (Nanjing, China).

### 4.6. Arabidopsis Transformation

The CDS sequence of each *VrYUCCA* gene was cloned into a pCAMBIA1305.1-GFP vector through XbaI and BamHI sites, to create a fusion construct under the control of the CaMV 35S promoter. The fusion vectors 35S::*VrYUCCAs*-GFP were transformed into *Agrobacterium tumefaciens* strain GV3101 and then were introduced into the *Arabidopsis thaliana* (Col-0) wild type (WT) using the floral dipping method described previously [51]. The seeds of transgenic *Arabidopsis* were screened on 1/2 MS culture media containing 15 mg/L hygromycin antibiotics. Phenotypic analysis was performed in the progeny-positive T_1_ lines.

## 5. Conclusions

In this study, 12 *VrYUCCAs* were systematically identified in mungbean, containing the six conserved motifs of the *YUCCA* gene family. The 12 *VrYUCCA* genes could be divided into four subfamilies according to the phylogenetic analysis. The *VrYUCCAs* showed different tissue expression patterns, and five had a highly specific transcript in the floral organs, including *VrYUCCA4b*, *VrYUCCA6a*, *VrYUCCA8b*, *VrYUCCA8c*, and *VrYUCCA11. VrYUCCA2a*, *VrYUCCA6b*, *VrYUCCA8a*, and *VrYUCCA10* seemed to regulate the IAA content throughout the whole growing period, due to the constitutive expression pattern in all the tissues. The VrYUCCA proteins participated in the IPA pathway for IAA synthesis in vitro and exhibited veritable biological activity to the IAA product in vivo, which was certified by 35S::*VrYUCCAs* transgenetic *Arabidopsis*. Five *YUCCAs* in the root responded to IAA, but only *VrYUCCA8a* responded in the leaf. Regarding the other four stresses, *VrYUCCA2a*, *VrYUCCA6a*, *VrYUCCA8a*, *VrYUCCA8b*, and *VrYUCCA10* might be mainly responsible for auxin regulation, due to their sensitivity to all four treatments used in this study.

## Data Availability

Not applicable.

## Supplementary Materials

The following supporting information can be downloaded at: https://www.mdpi.com/article/10.3390/ijms24021603/s1.
