# Supplementary material for: Genome-Wide Identification, Expression Analysis, and Potential Roles under Abiotic Stress of the YUCCA Gene Family in Mungbean (Vigna radiata L.)
Source: Int J Mol Sci. 2023 Jan 13;24(2):1603. doi: 10.3390/ijms24021603 (PMC9866024; doi:10.3390/ijms24021603)
Supplement: Supplementary file 1 [file ijms-24-01603-s001.zip › supplement figures.pdf]

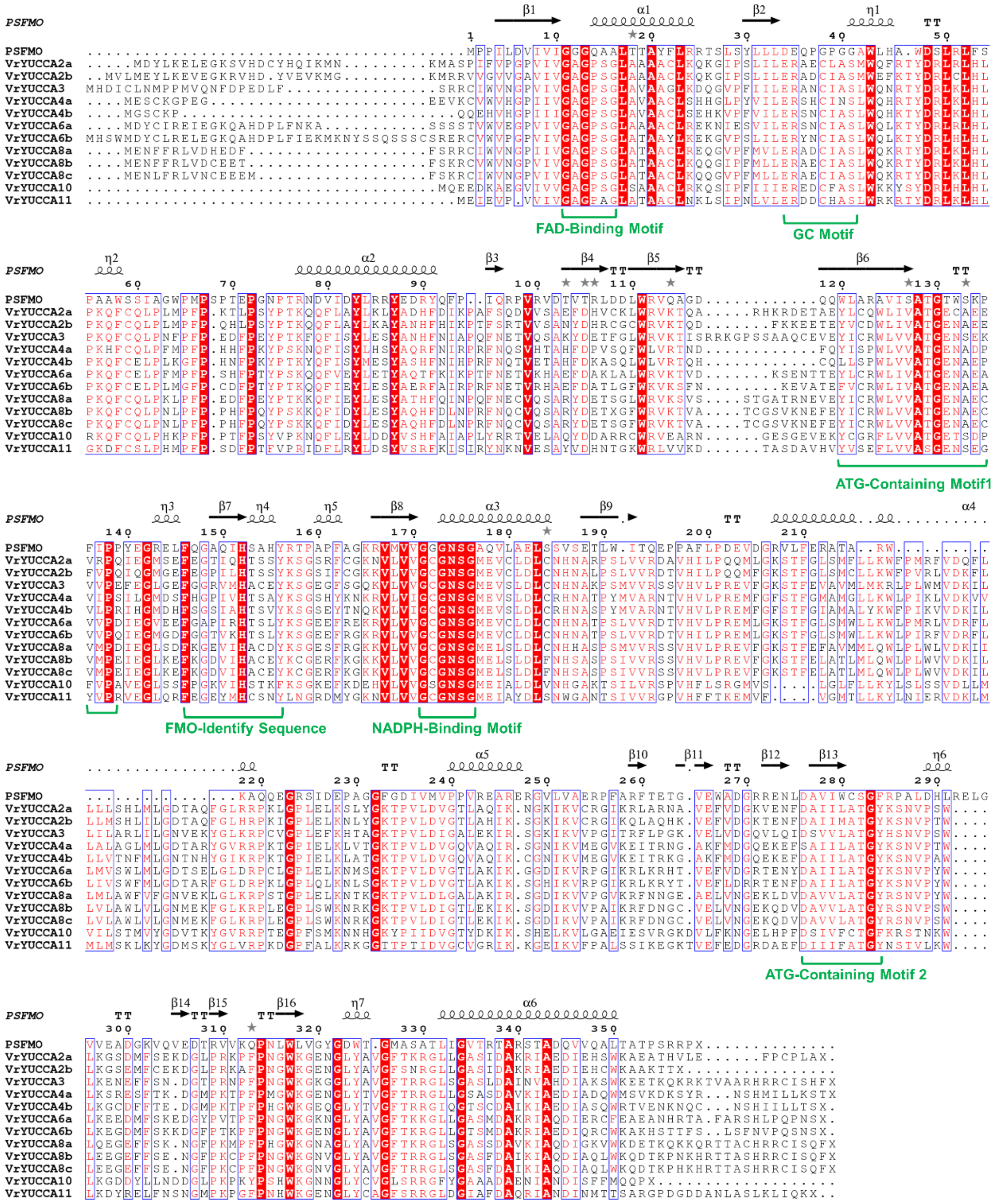

Figure. S1. Sequence alignment of 12 VrYUCCA proteins. PSFMO is a flavin-containing monooxygenase (FMO) from *Pseudomonas stutzeri* NF13 and its structure has been clarified. The conserved FAD binding motif, GC motif, ATG-containing motif 1, FMO-identifying sequence, NADPH-binding motif, and ATG-containing motif 2 are marked by green lines. Alternate residues are highlighted by gray stars.

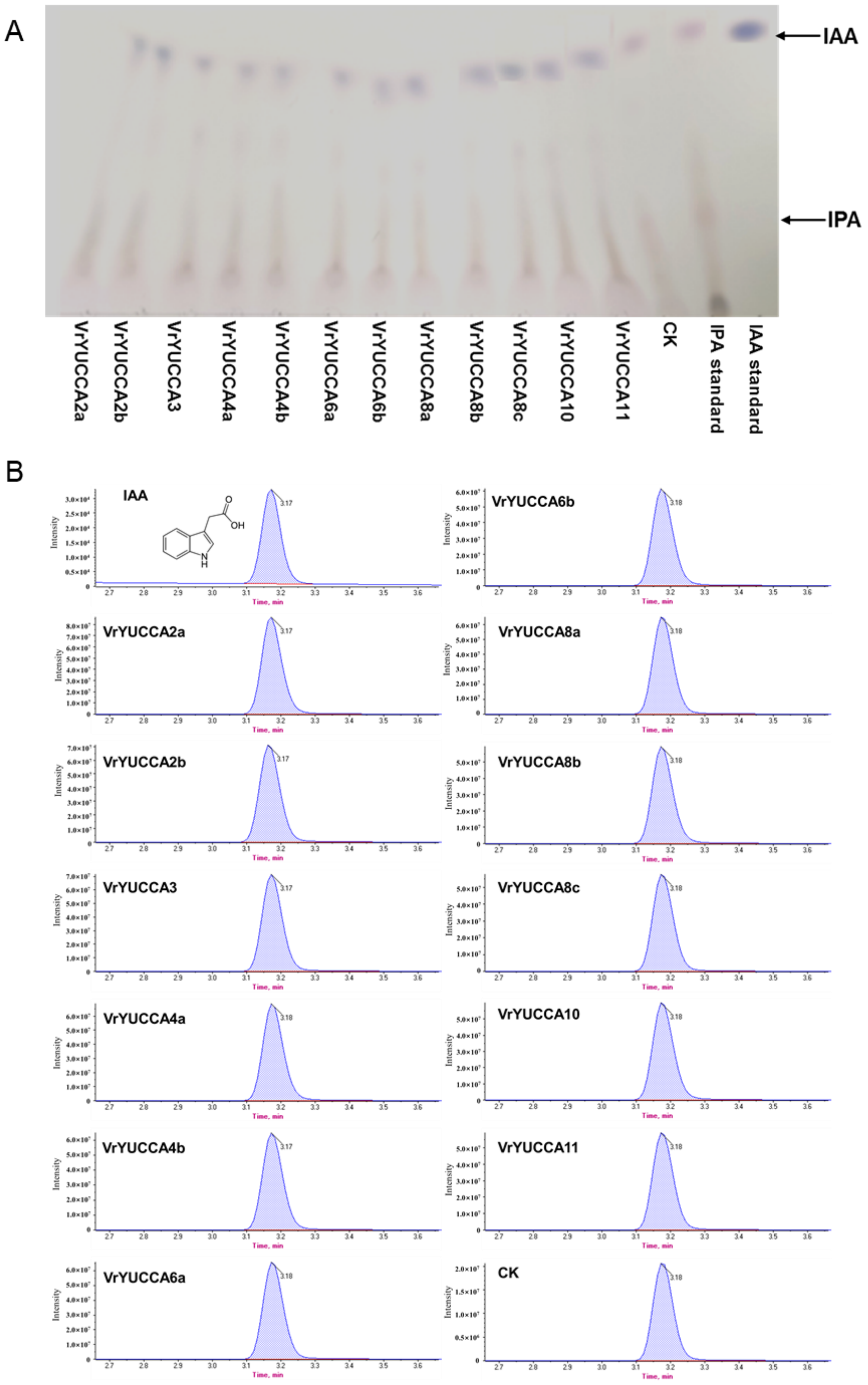

Figure. S2. Enzymatic assays of VrYUCCAs toward the substrates IPA analyzed by TLC (A) and HPLC-MS/MS (B).

(A) Enzymatic products were displayed by TLC. The coloration of IPA on the TLC plate was light pink, while that of IAA was blue-purple. The position of standard IPA and IAA have marked by the arrows. A small amount of IAA could be detected in CK line due to spontaneous conversion of IPA to IAA at room temperature. (B) The IAA content in reaction mixtures was detected by HPLC-MS/MS. CK: the enzyme reaction mixture containing a boiled protein. The relatively lower HPLC profile in CK was caused by spontaneous conversion of IPA to IAA.

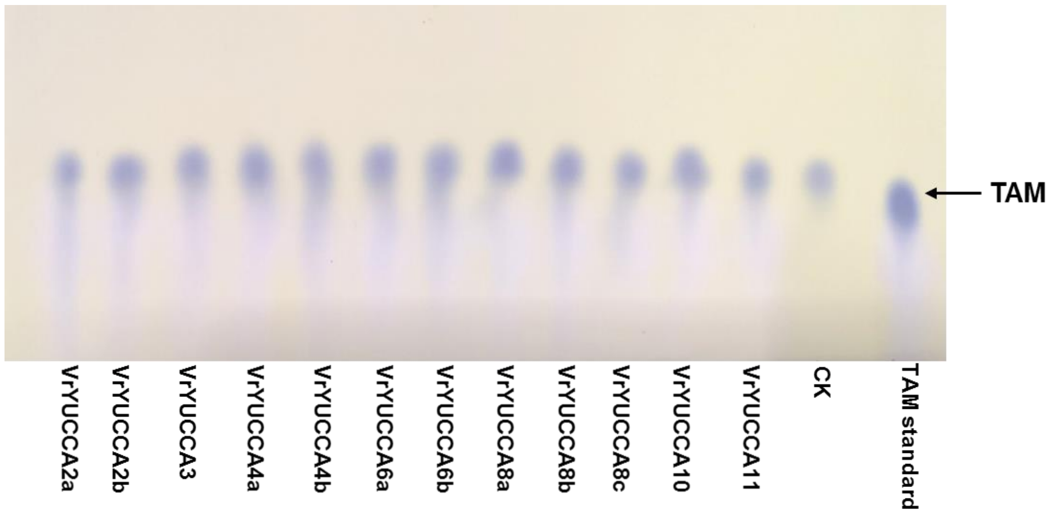

Figure. S3. Enzymatic assays of VrYUCCAs toward the substrates TAM analyzed by TLC.

No new chemical compound was produced as exhibited on the TLC plate. The product of this reaction was assumed as N-hydroxytryptamine, and the standard was hardly obteined from current chemical reagents corporation. So the standard of N-hydroxytryptamine did not be shown on the TLC plate.

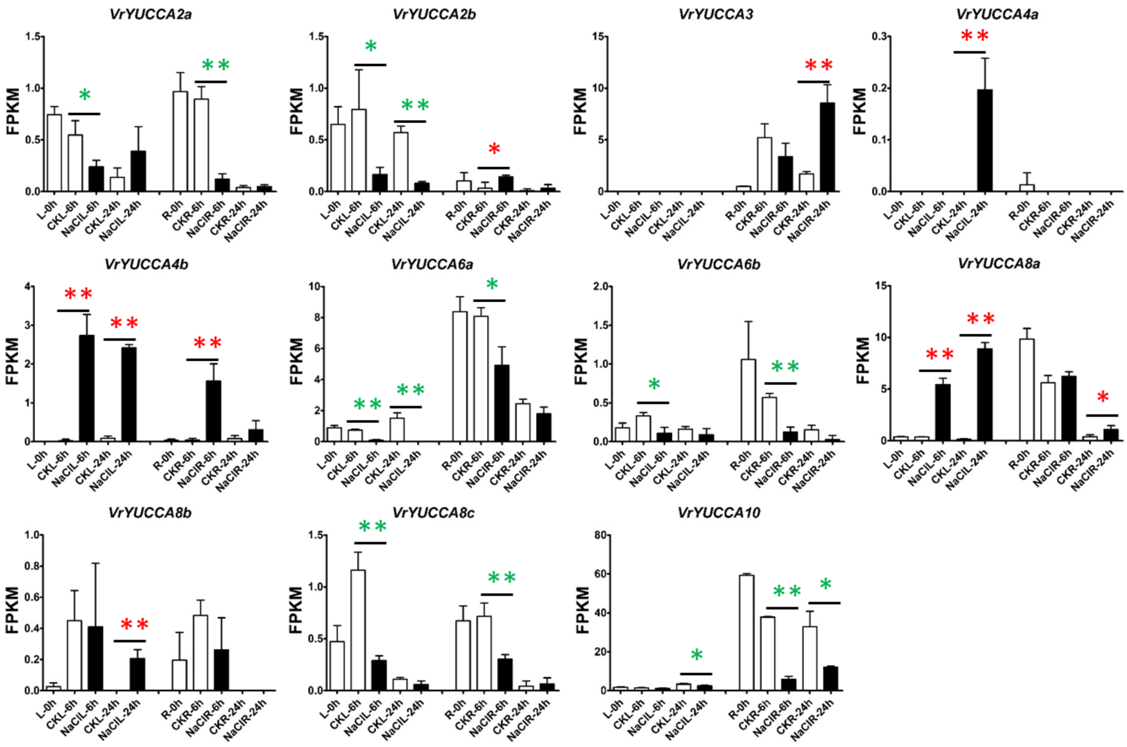

Figure. S4. Effect of high salinity on the expression of *VrYUCCAs*.

FPKM values were obtained from the result of RNA-seq. The difference significance analysis was performed between each treatment and its contemporaneous control group(CK). L: leaf; R: root. The green stars mean the expression of *VrYUCCAs* declined compared with CK, and the red stars mean the expression increased. *, $p<0.05$; **, $p<0.01$. Error bars indicated SDs (standard deviations) from three biological repetitions.

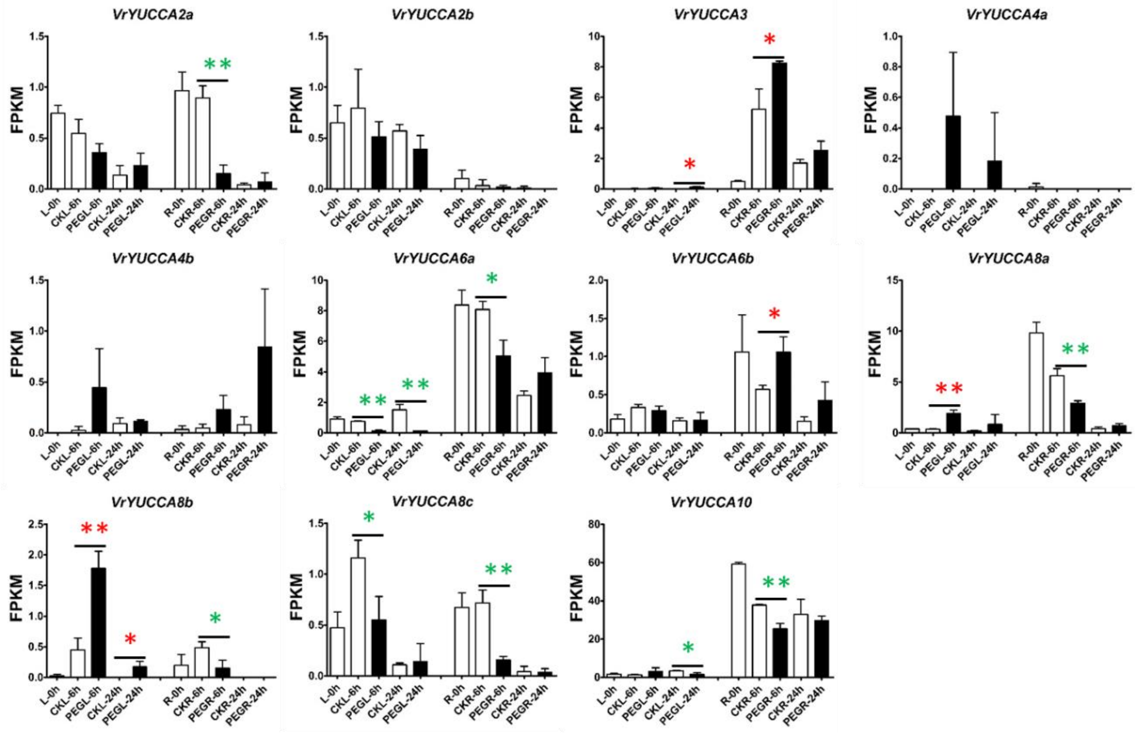

Figure. S5. Effect of drought on the expression of *VrYUCCAs*.

FPKM values were obtained from the result of RNA-seq. The difference significance analysis was performed between each treatment and its contemporaneous control group (CK). L: leaf; R: root. The green stars mean the expression of *VrYUCCAs* declined compared with CK, and the red stars mean the expression increased. *, $p<0.05$; **, $p<0.01$. Error bars indicated SDs from three biological repetitions.

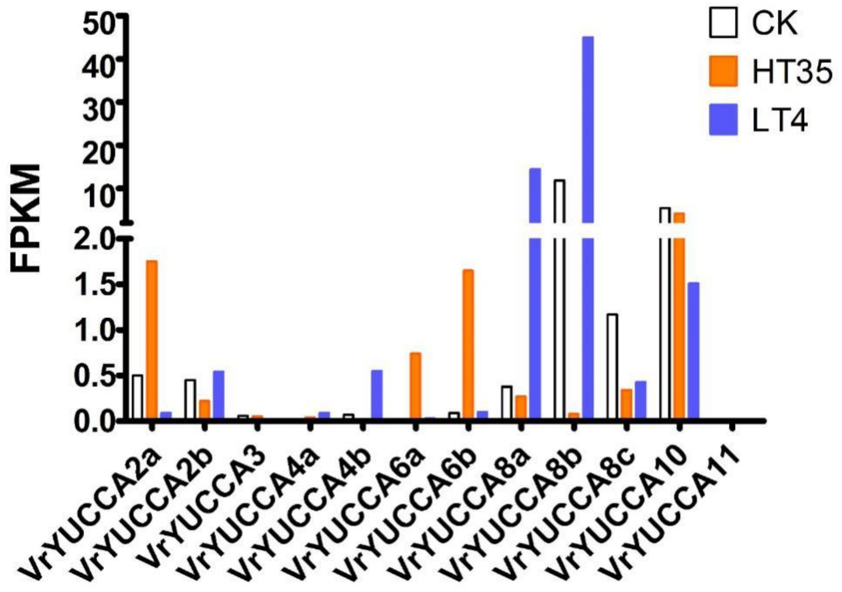

Figure. S6. Effect of high and low temperature on the expression of *VrYUCCAs.*
FPKM values were obtained from the result of RNA-seq. CK: the seedlings grown under 25°C; HT35: the seedlings grown under high temperatures, 35°C; LT4, the seedlings grown under low temperatures, 4°C.
