# Supplementary material for: Genome-Wide Identification, Expression Analysis, and Potential Roles under Abiotic Stress of the YUCCA Gene Family in Mungbean (Vigna radiata L.)
Source: Int J Mol Sci. 2023 Jan 13;24(2):1603. doi: 10.3390/ijms24021603 (PMC9866024; doi:10.3390/ijms24021603)
Supplement: Supplementary file 1 [file ijms-24-01603-s001.zip › supplementary data S1- sequence information.pdf]

Supplementary Data S1. Predicted amino acid sequence of VrYUCCAs, AtYUCCAs, GmYUCCAs, OsYUCCAs and MtYUCCAs.

1. VrYUCCAs

(1) VrYUCCA2a(LOC106767860)

MDYLKELEGKSVHDCYHQIKMNKMASPIFVPGPVIVGAGPSGLAAAACLKQKGIPSLILE
RAECLASMWQFRTYDRLRLHLPKQFCQLPLMPFPKTLPSYPTKQQFLAYLKLYADHFDI
KPAFSQDVVSAEFDHVCKLWRVKTQARHKRDETAEYLCQWLIVATGECAEEVRPQIEG
MSEFEGTIQHTSSYKSGSRFCGKNVLVVGCGNSGMEVCLDLCNHNARPSLVVRDAVHI
LPQQMLGKSTFGLSMFLLKWFPMRFVDQFLLLLSHLMLGDTAQFGLRRPKLGPLELKSL
YGKTPVLDVGTLAQIKNGKIKVCRGIKRLARNAVEFVDGKVENFDAIILATGYKSNVPSWL
KGSDMFSEKDGLPRKPFPNGWKGENGLYAVGFTKRGLLGASIDAKRIAEDIEHSWKAEA
THVLEFPCPLA*

(2) VrYUCCA2b(LOC106767822)

MVLMEYLKEVEGKRVHDYVEVKMGKMRRVVGVVGAVIVGAGPSGLAAAACLKQKGIPS
LILERADCIASMWEFKTYDRLCLHLPKQFCQLPLMPFPQHLPSYPTKQQFLAYLKAYANH
FHIKPTFSRTVVSANYDHRCGCWRVKTQDFKKEETEYVCDWLIVATGENAEEFVPQIQG
MGEFEGPILHTSSYKSGSIFCGKKVLVVGCGNSGMEVCLDLCNHNALPSLVVRDTVHILP
QQMFGKSTFGLSMCLLKWFPVRLVDKFLLLMSHLILGDTAQFGLHRPKIGPLELKNLYGK
TPVLDVGTLAHIKSGKIKVCRGIKQLAQHKVEFVDGKTENFDAIIMATGYKSNVPTWLKG
SEMFCEKDGLPRKAFPNGWKGENGLYAVGFSNRGLLGASIDAKRIAEDIEHCWKAAKTT
*

(3) VrYUCCA3(LOC106770152)

MHDICLNMPPMVQNFDPEDLFSRRCIWVNGPVIVGAGPSGLAVAAGLKDQGVPFIILER
ANCIASLWQNRTYDRLKLHLPKQFCQLPNFPFPEDFPEYPTKFQFISYLESYANHFNIAP
QFNETVQSAKYDETFGLWRVKTISRRKGPSSAAQCEVEYICRWLVVATGENSEKVVPEF
EGLGEFGGRVMHACEYKSGEGFSGQKVLVVGCGNSGMEVSLDLCNHNAKPSMVVRS
SVHVLPREVFGKSTFEVAVMLMKRLPLWMVDKILLILARLILGNVEKYGLKRPCVGPLEFK
HTAGKTPVLDIGALEKIRSGKIKVVPGITRFLPGKVELVDGQVLQIDSVVLATGYHSNVPS
WLKENEFFSNDGTPRNPFPNGWKGKGGLYAVGFTRRGLSGASLDAINVAHDIAKSWKE
ETKQKRKTVAARHRRCISHF*

(4) VrYUCCA4a(LOC106761746)

MESCKGPEGEEVKCVWVHGPIIVGAGPSGLAVAACLSHHGLPYVILERSHCINSLWQHR
TYDRLKLHLPKHFCQLPFMPFPHHFPKYPSKNQFISYLHSYAQRFNIRPRFNQSVHTAHF
DPVSQFWLVRTNDFQYISPWLVVATGENADPVIPSILGMDSFHGPIVHTSAYKSGSHYKN
KRVLVIGCGNSGMEVSLDLCRHNATPYMVARNTVHVLPREMFGFSTFGMAMGLLKWLP
IKLVDKVVLALAGLMLGDTARYGVRRPKTGPIELKLVTGKTPVLDVGQVAQIRSGNIKVME
GVKEITRNGAKFMDGQEKEFSAIILATGYKSNVPTWLKSRESFTKDGMPKTPFPMGWK
GENGLYTVGFTRRGLLGSASDAVKIAEDIADQWMSVKDKSYRNSHMILLKST*

(5) VrYUCCA4b(LOC106764471)
MGSCKPQQEHVHGPIIIGAGPSGLAVAACLSEHKVPFVILERSNCIASLWQHKTYDRLKL
HLPKQFCELPLKGFPHNFPKYPTKYQFISYMESYASHFNIHPRFNQTVETAHFDKASQL
WLVRTQHCQLLSPWLVVATGENAEPVLPRIHGMDHFSGSIAHTSVYKSGSEYTNQKVLV
IGCGNSGMEVSLDLCRHNASPYMVARNTVHVLPREMFGFSTFGIAMALYKWFPIKVVDK
ILLLVTNFMLGNTNHYGIKRPKTGPIELKLATGKTPVLDVGQVAQIKCGNIKVMEGVKEITR
KGAKFMDGQEKEFDAIILATGYKSNVPAWLKGCDFFTEDGMPKTPFPHGWKGEQGLYT
VGFTRRGIQGTSCDAIKIAEDIASQWRTVENKNQCNSHIILLTS*

(6) VrYUCCA6a(LOC106761281)
MDYCIREIEGKQAHDPLFNKASSSSTVWVEGPVIVGAGPSGLAAAACLREKNIESVILER
SNCIASLWQLKTYDRLRLHLPKQFCELPFMPFPSHFPTYPSKQQFVEYLETYAQTFKIKP
TFNETVKHAEFDAKLALWRVKTVDKSENTTEYLCRWLIVATGENAEAVVPDIEGVEEFGA
PIRHTSLYKSGEEFREKRVLVVGCGNSGMEVCLDLCNHNATPSLVVRDTVHVLPREMLG
KSTFGLSMWLLKWLPMRLVDRFLLMVSWLMLGDTSELGLDRPCLGPLELKNMSGKTPV
LDVGTLAKIKGGDIKVRPGIKRLKRHTVEFVDGRTENYDAIILATGYKSNVPYWLKEEDMF
SKEDGYPVTPFPNGWKGKNGLYAVGFTKKGLLGASMDAKRIAQDIERCFEAEANHRTAF
ARSHLPQPNS*

(7) VrYUCCA6b(LOC106764337)
MHSWMDYCLRELEGKQAHDPLFIEKMKNYSSQSSSCSRERCVWVPGPVIVGAGPSGL
ATAAYLKEKGVPSLILERYNCIASLWQLKTYDRLHLHLPKQFCELPLMGFPCDFPTYPTK
QQFIEYLESYAERFAIRPRFNETVRHAEFDATLGFWKVKSFNKEVATEFVCRWLIVATGE
NAEAVVPQIEGMGDFGGTVKHTSLYKSGEEFRGKRVLVVGCGNSGMEVCLDLCNHNAT
PSLVVRDTVHILPREMLGKSTFGLSMWLLKWLPIRFVDRFLLIVSWFMLGDTARFGLDRP
KLGPLQLKNLSGKTPVLDVGTLAKIKSGHIKVRPGIKRLKRYTVEFLDRRTENFDAIILATG
YKSNVPYWLKEGDMFSKKDGFPTKPFPNGWKGENGLYAVGFTKRGLLGTSMDAKRIAE
DIQRCWKAKHSTTFSLSFNVPQSNS*

(8) VrYUCCA8a(LOC106757377)
MENFFRLVDHEDFFSRRCIWVNGPIIVGAGPSGLATAACLREQGVPFMVLERADCIASL
WQKRTYDRLKLHLPKQFCQLPKLPFPEDFPEYPTKKQFIEYLESYATHFQINPQFNECVQ
SARYDETSGLWRVKSVSSTGATRNEVEYICRWLVVATGENAECVMPDIEGLSDFKGEVI
HACDYKSGESFRGKKVLVVGCGNSGMELSLDLCNHHASPSMVVRSSVHVLPREVFGK
STFEFAVMMLQWLPLWLVDKIILMLAWFVFGNVEKLGLKRPSTGPLELKNTKGKTPVLDL
GALAKIRSGDIKVVPGVKRFNNGEAELVNGEKLDVDAVVLATGYRSNVPYWLQEGEFFS
KNGFPKMPFPHGWKGNAGLYAVGFTKRGLSGASSDAVKIAQDIGKVWKDETKQKKQRT
TACHRRCISQF*

(9) VrYUCCA8b(LOC106771549)
MENFFRLVDCEETFSKRCVWVNGPVIVGAGPSGLATAACLKQQGIPFMLLERAECIASL
WQKRTYDRLKLHLPKQFCQLPNLPFPPHFPQYPSKKQFIDYLESYAQHFDLNPRFNQCV
QSARYDETXGFWRVKTVATCGSVKNEFEYICRWLVVATGENAECVMPEIEGLKEFKGDV
IHACEYKCGERFKGKKVLVVGCGNSGMELSLDLFNHSASPSIVVRSSVHVLPREVFGKS

TFELATLMLQWLPLWVVDKILLVLAWLVLGNMEKFGLKRPLEGPLSWKNRKGKTPVLDIG
TLEKIKSGDIKVVPAIKRFDNGCVELVNGEKQDVDAVVLATGYRSNVPSWLEEGEFFSEN
GFPKCPFPNGWKGNVGLYAVGFTRRGLSGASFDAIKIAQDIAQLWKQDTKPNKHRTTAS
HRRCISQF*

(10) VrYUCCA8c(LOC106771284)
MENLFRLVNCEEEMFSKRCIWVNGPVIVGAGPSGLATAACLKQQGVPFMLLERAECIAS
LWQKRTYDRLKLHLPKQFCQLPNLPFPPHFPQYPSKKQFIDYLESYAQHFDLNPRFNQC
VQSARYDETSGFWRVKTVATCGSVKNEFEYICRWLVVATGENAECVMPEIEGLKEFKGD
VIHACEYKCGERFKGKKVLVVGCGNSGMELSLDLFNHSASPSIVVRSSVHVLPREVFGK
STFELATLMLQWLPLWVVDKILLVLAWLVLGNMEKFGLKRPLEGPLSWKNRKGKTPVLDI
GTLEKIKSGDIKVVPAIKRFDNGCVELVNGEKQDVDAVVLATGYRSNVPSWLEEGEFFSE
NGFPKCPFPNGWKGNVGLYAVGFTRRGLSGASFDAIKIAQDIAQLWKQDTKPHKHRTTA
SHRRCISQF*

(11) VrYUCCA10(LOC106757305)
MQEEDKAEGVIVVGAGPSGLSAAACLRKQSIPFIIIEREDCFASLWKKYSYDRLHLHLRK
QFCQLPHKPFPPTFPSYVPKNQFLEYLDDYVSHFAIAPLYRRTVELAQYDDARRCWRVE
ARNGESGEVEKYCGRFLVVATGETSDPFVPAVEGLSSFPGKVIHSTKFKSGKEFKDEHV
LVVGSGNSGMEIALDLVNHGAKTSILVRSPVHFLSRGMVSLGLFLLKYLSLSSVDLLMVIL
STMVYGDVTKYGVRRPTEGPFSMKNNHGKYPIIDVGTYDKIKSHELKVLGAEIESVRGK
DVLFKNGELHPFDSIVFCTGFKRSTNKWLKGDDYLLNDDGLPKPKYPSHWKGNNGLYC
VGLSRRGFYGAAADAENIANDISFFMQQP*

(12) VrYUCCA11(LOC106778822)
MEIEVPVVIVGAGPAGLATAACLNKLSIPNLVLERDDCHASLWRKRTYDRLKLHLGKDFC
SLPHMPFPSDFPTFVPRIDFLRYLDSYVSRFKISIRYNKNVESAYVDHNTGKWRLVVKDT
ASDAVHVYVSEFLVVASGENSEGYVPRVEGLQRFEGEYMHCSNYLNGRDMYGKNVLV
VGCGNSGMEIAYDLSNWGANTSIVVRGPVHFFTKEMVFVGMTLLKYLNIERVDKLMML
MSKLKYGDMSKYGLVRPKDGPFALKRKGGTTPTIDVGCVGRIKKGEIKVFPALSSIKEGK
TVEFEDGRDAEFDIIIFATGYNSTVLKWLKDYRELFNSNGMPKPGFPNHWKGENGLYCA
GFSRRGLDGIAFDAQRIANDINMTTSARGPDGDDANLASLKLIQK*

2. AtYUCCAs

(1) AtYUCCA1(At4g32540)
MESHPHNKTDQTQHIILVHGPIIIGAGPSGLATSACLSSRGVPSLILERSDSIASLWKSKTY
DRLRLHLPKHFCRLPLLDFPEYYPKYPSKNEFLAYLESYASHFRIAPRFNKNVQNAAYDS
SSGFWRVKTHDNTEYLSKWLIVATGENADPYFPEIPGRKKFSGGKIVHASEYKSGEEFR
RQKVLVVGCGNSGMEISLDLVRHNASPHLVVRNTVHVLPREILGVSTFGVGMTLLKCLPL
RLVDKFLLLMANLSFGNTDRLGLRRPKTGPLELKNVTGKSPVLDVGAMSLIRSGMIQIME
GVKEITKKGAKFMDGQEKDFDSIIFATGYKSNVPTWLQGGDFFTDDGMPKTPFPNGWR
GGKGLYTVGFTRRGLLGTASDAVKIAGEIGDQWRDEIKGSTRNMCSSRFVFTSKS*

(2) AtYUCCA2(At4g13260)

MEFVTETLGKRIHDPYVEETRCLMIPGPIIVGSGPSGLATAACLKSRDIPSLILERSTCIASL
WQHKTYDRLRLHLPKDFCELPLMPFPSSYPTYPTKQQFVQYLESYAEHFDLKPVFNQTV
EEAKFDRRCGLWRVRTTGGKKDETMEYVSRWLVVATGENAEEVMPEIDGIPDFGGPILH
TSSYKSGEIFSEKKILVVGCGNSGMEVCLDLCNFNALPSLVVRDSVHVLPQEMLGISTFG
ISTSLLKWFPVHVVDRFLLRMSRLVLGDTDRLGLVRPKLGPLERKIKCGKTPVLDVGTLA
KIRSGHIKVYPELKRVMHYSAEFVDGRVDNFDAIILATGYKSNVPMWLKGVNMFSEKDG
FPHKPFPNGWKGESGLYAVGFTKLGLLGAAIDAKKIAEDIEVQRHFLPLARPQHC*

(3) AtYUCCA3(At1g04610)
MYGNNNKKSINITSMFQNLIPEGSDIFSRRCIWVNGPVIVGAGPSGLAVAAGLKREGVPFI
ILERANCIASLWQNRTYDRLKLHLPKQFCQLPNYPFPDEFPEYPTKFQFIQYLESYAANF
DINPKFNETVQSAKYDETFGLWRVKTISNMGQLGSCEFEYICRWIVVATGENAEKVVPDF
EGLEDFGGDVLHAGDYKSGGRYQGKKVLVVGCGNSGMEVSLDLYNHGANPSMVVRS
AVHVLPREIFGKSTFELGVTMMKYMPVWLADKTILFLARIILGNTDKYGLKRPKIGPLELK
NKEGKTPVLDIGALPKIRSGKIKIVPGIIKFGKGKVELIDGRVLEIDSVILATGYRSNVPSWL
KDNDFFSDDGIPKNPFPNGWKGEAGLYAVGFTRKGLFGASLDAMSVAHDIANRWKEES
KQQKKTAAARHRRCISHF*

(4) AtYUCCA4(At5g11320)
MGTCRESEPTQIFVPGPIIVGAGPSGLAVAACLSNRGVPSVILERTDCLASLWQKRTYDR
LKLHLPKHFCELPLMPFPKNFPKYPSKQLFISYVESYAARFNIKPVFNQTVEKAEFDDAS
GLWNVKTQDGVYTSTWLVVATGENAEPVFPNIPGLKKFTGPVVHTSAYKSGSAFANRKV
LVVGCGNSGMEVSLDLCRYNALPHMVVRNSVHVLPRDFFGLSTFGIAMTLLKWFPLKLV
DKFLLLLANSTLGNTDLLGLRRPKTGPIELKNVTGKTPVLDVGAISLIRSGQIKVTQAVKEI
TRNGAKFLNGKEIEFDSIILATGYKSNVPDWLKENSFFTKEGMPKTPFFPNGWKGEKGLY
TVGFTRRGLSGTAYDAVKIAEDITDQWMKFNGPLSCRNICSSHIIHLHFNKS*

(5) AtYUCCA5(At5g43890)
MENMFRLMGSEDSSDRRRCIWVNGPVIVGAGPSGLATAACLREEGVPFVVLERADCIA
SLWQKRTYDRIKLHLPKKVCQLPKMPFPEDYPEYPTKRQFIEYLESYANKFEITPQFNEC
VQSARYDETSGLWRIKTTSSSSSGSEMEYICRWLVVATGENAEKVVPEIDGLTTEFEGEV
IHSCEYKSGEKYRGKSVLVVGCGNSGMEVSLDLANHNANASMVVRSSVHVLPREILGK
SSFEISMMLMKWFPLWLVDKILLILAWLILGNLTKYGLKRPTMGPMELKIVSGKTPVLDIG
AMEKIKSGEVEIVPGIKRFSRSHVELVDGQRLDLDAVVLATGYRSNVPSWLQENDLFSK
NGFPKSPFPNAWKGKSGLYAAGFTRKGLAGASADAVNIAQDIGNVWREETKRQKMRTR
VGHRRCISVA*

(6) AtYUCCA6(At5g25620)
MDFCWKREMEGKLAHDHRGMTSPRRICVVTGPVIVGAGPSGLATAACLKERGITSVLLE
RSNCIASLWQLKTYDRLHLHLPKQFCELPIIPFPGDFPTYPTKQQFIEYLEDYARRFDIKP
EFNQTVESAAFDENLGMWRVTSVGEEGTTEYVCRWLVAATGENAEPVVPRFEGMDKF
AAAGVVKHTCHYKTGGDFAGKRVLVVGCGNSGMEVCLDLCNFGAQPSLVVRDAVHVLP
REMLGTSTFGLSMFLLKWLPIRLVDRFLLVVSRFILGDTTLLGLNRPRLGPLELKNISGKT
PVLDVGTLAKIKTGDIKVCSGIRRLKRHEVEFDNGKTERFDAIILATGYKSNVPSWLKENK

MFSKKDGFPIQEFPEGWRGECGLYAVGFTKRGISGASMDAKRIAEDIHKCWKQDEQVK
KI*

(7) AtYUCCA7(At2g33230)
MCNNNNTSCVNISSMLQPEDIFSRRCIWVNGPVIVGAGPSGLAVAADLKRQEVPFVILER
ANCIASLWQNRTYDRLKLHLPKQFCQLPNLPFPEDIPEYPTKYQFIEYLESYATHFDLRPK
FNETVQSAKYDKRFGLWRVQTVLRSELLGYCEFEYICRWLVVATGENAEKVVPEFEGLE
DFGGDVLHAGDYKSGERYRGKRVLVVGCGNSGMEVSLDLCNHDASPSMVVRSSVHVL
PREVLGKSTFELSVTMMKWMPVWLVDKTLLVLTRLLLGNTDKYGLKRPEIGPLELKNTA
GKTPVLDIGAISMIKSGKIKIVAGIAKFGPGKVELVDGRVLQIDSVILATGYRSNVPSWLKE
NDLGEIGIEKNPFPKGWKGKAGLYAVGFTGRGLSGASFDAMSVAHDIANSWKEETKQQI
KTVATRHRRCISHF*

(8) AtYUCCA8(At4g28720)
MENMFRLMDQDQDLTNNRCIWVNGPVIVGAGPSGLATAACLHEQNVPFVVLERADCIA
SLWQKRTYDRLKLHLPKQFCQLPKMPFPEDFPEYPTKRQFIDYLESYATRFEINPKFNEC
VQTARFDETSGLWRVKTVSKSESTQTEVEYICRWLVVATGENAERVMPEIDGLSEFSGE
VIHACDYKSGEKFAGKKVLVVGCGNSGMEVSLDLANHFAKPSMVVRSSLHVMPREVMG
KSTFELAMKMLRWFPLWLVDKILLVLSWMVLGNIEKYGLKRPEMGPMELKSVKGKTPVL
DIGAIEKIRLGKINVVPGIKRFNGNKVELVNGEQLDVDSVVLATGYRSNVPYWLQENEFF
AKNGFPKTVADNNGWKGRTGLYAVGFTRKGLSGASMDAVKIAQDIGSVWQLETKQPTK
RSRGSLRRCISQQF*

(9) AtYUCCA9(At1g04180)
MENMFRLMASEEYFSERRCVWVNGPVIVGAGPSGLATAACLHDQGVPFVVVERSDCIA
SLWQKRTYDRLKLHLPKKFCQLPKMPFPDHYPEYPTKRQFIDYLESYANRFDIKPEFNKS
VESARFDETSGLWRVRTTSDGEEMEYICRWLVVATGENAERVVPEINGLMTEFDGEVIH
ACEYKSGEKFRGKRVLVVGCGNSGMEVSLDLANHNAITSMVVRSSVHVLPREIMGKST
FGISVMMMKWLPLWLVDKLLLILSWLVLGSLSNYGLKRPDIGPMELKSMTGKTPVLDIGA
LEKIKSGDVEIVPAIKQFSRHHVELVDGQKLDIDAVVLATGYRSNVPSWLQESEFFSKNG
FPKSPFPNAWKGKSGLYAAGFTRKGLAGASVDAVNIAQDIGNVWREETKRQKMRRNVG
HRRCISVA*

(10) AtYUCCA10(At1g48910)
METVVVIVGAGPAGLATSVCLNQHSIPNVILEKEDIYASLWKKRAYDRLKLHLAKEFCQLP
FMPHGREVPTFMSKELFVNYLDAYVARFDINPRYNRTVKSSTFDESNNKWRVVAENTVT
GETEVYWSEFLVVATGENGDGNIPMVEGIDTFGGEIMHSSEYKSGRDFKDKNVLVVGG
GNSGMEISFDLCNFGANTTILIRTPRHVVTKEVIHLGMTLLKYAPVAMVDTLVTTMAKILYG
DLSKYGLFRPKQGPFATKLFTGKAPVIDVGTVEKIRDGEIQVINGGIGSINGKTLTFENGH
KQDFDAIVFATGYKSSVCNWLEDYEYVMKKDGFPKAPMPKHWKGEKNLYCAGFSRKGI
AGGAEDAMSVADDIRSILATLKNN*

(11) AtYUCCA11(At1g21430)
MEKEIKILVLIIGAGPAGLATSACLNRLNIPNIVVERDVCSASLWKRRSYDRLKLHLAKQFC

QLPHMPFFPSNTPTFVSKLGFINYLDEYATRFNVNPRYNRNVKSAYFKDGQWIVKVVNKT
TALIEVYSAKFMVAATGENGEGVIPEIPGLVESFQGKYLHSSEYKNGEKFAGKDVLVVGC
GNSGMEIAYDLSKCNANVSIVVRSQVHVLTRCIVRIGMSLLRFFPVKLVDRLCLLLAELRF
RNTSRYGLVRPNNGPFLNKLITGRSATIDVGCVGEIKSGKIQVVTSIKRIEGKTVEFIDGNT
KNVDSIVFATGYKSSVSKWLEVDDGDLFNENGMPKREFPDHWKGKNGLYSAGFGKQG
LAGISRDARNIARDIDSLVCGRSSKNKLSK*

3. GmYUCCAs

(1) GmYUCCA1(GLYMA_03G169600)
MENLFRLVDNEDLFSRRCIWVNGPIIVGAGPSGLATAACLREQGVPFMVLERADCIASLW
QKRTYDRLKLHLPKQFCQLPKLPFPEDFPEYPTKKQFIEYLESYAKHFEINPQFNECVQS
ARYDETSGLWRVKTVSSSSGAARGEVEYICRWLVVATGENAECVMPEIEGLSEFKGDVI
HACDYKSGERFRGKKVLVVGCGNSGMELSLDLCNHHSSPSMVVRSSVHVLPREVFGIS
TFELAVMLLQWLPLWLVDKILLILAWFVLGNIEKLGLKRPSKGPLEMKNRKGKTPVLDIGA
LERIRSGDIEVVPGIKRFNNGEVEFINGEKLDIDAIVLATGYRSNVPSWLQEGEFFSKNGY
PKMPFPHGWKGNAGLYAVGFTKRGLSGASSDAVKIAQDIGQVWKNETKQKKQRTTACH
RRCISQF*

(2) GmYUCCA2(GLYMA_03G208900)
MEEKQRVIIVGAGPSGISAAACFTKQSIPYIILEREDCSASLWKKYSYERLHLHLRKQYCQ
LPHKPFPASFPPYVPKKQFLQYLDDYVSHFGITPLYRRTVELAEYDQGCHNWRVMALNG
DSGQLEEYRGRFLVVATGETTDPFVPELQGLSGFPGKLIHSTGFRSGKDFKDQHVLVVG
SGNSGMEIALDLVNHGAKTSILVRSPVHFLSREMVSLGLFLLKYLSLSTVDSLMVMLSTMI
YGDVTKYGVARPNEGPFYMKVKYGKYPVIDVGTYKKIKSGELKVLPSEIESLRGKDVLFK
NGESHPFDSIVFCTGFKRSTNKWLKGDDYLLNDEGLPKPSYPIHWKGNNGLYCVGLSR
RGFYGAAADAENIANDVSSFTQHPCP*

(3) GmYUCCA3(GLYMA_04G213600)
MEEVSVIIVGAGPSGLAISACLSQNFISHIILEKEDCSASLWRKNAYDRLKLHLASEFCVLP
LMPHPPSAPTYLSKDEFVQYIDSYIACFNINPLYCRMVEFAAYDEVENKWRVEAKKTLEG
TSETYVAKFLVIATGENSEGYIPDVPGLESFEGEIVHSKYYKSGSKYETKEVLVVGCGNS
GMEIAYDLNDWGANTSILIRNPVHVFTKELINEGMRMLKHLPVHVVDNIITSLANMEYGDL
SKYGIYQPKKGPFHLKFITGRAPVIDVGTIEKIKEGAIKVIPSHIVRIENKKVIFENDAEKEFD
VIVFATGYRSVANKWLKDYKYVLNDEGMPNNDFPNHWKGDRGLYCAGLSNRGLFGVK
MDVEAIADDINQTLKLH*

(4) GmYUCCA4(GLYMA_04G242500)
MNSQAYKSSTQQREENHQSHCITNLKPKHQNQTMEIEVPVVIVGAGPAGLATAACLNKY
SIPNVVLERHDCHASLWRKRTYDRLKLHLGKDFCNLPHMPFPLDFPTFVPRVDFLRYLD
NYVTRFKISIRYTRNVESASVDEENNGKWRVVVKDTTTNADEVYVADYLVVATGENDEG
YVPQIEGLEGFEGEHMHCSQYLNGRHLYGKNVLVVGSGNSGMEIAYDLSTWGANTSIVI
RGPVHYFTKEMVFVGMSLLKYFKMEKVDKLMLLMSKLKYGDMSEYGLVRPKDGPFFLKI
KGGTTPTIDVGCVSRIKKGEVKVFPAISSIKKDKMVEFEDGQNGQFDVIIFATGYNSTVLK
WLKDYRGLFNENGMPKPSFPNHWKGNNGIYCAGFSRRGLDGIAFDAQRIADDIKKTLN

ARNLPGDEEDNFASLKLLGK*

(5) GmYUCCA5(GLYMA_04G070100)
MESLKGEEVKCVWVQGPIIVGAGPSGLAVAACLSHHGVPYVILERSHCITSLWQHRTYD
RLKLHLPKHFCELPLMPFPLHFPKYPSKNQFISYLNSYASRFNIRPRFNQSVQTAEFDPS
SQLWLVRTNGFQYISPWLVVATGENAEPVVPSISGMDMFHGPIVHTSVYKSGSDYNNQR
VLVIGCGNSGMEVSLDLCRHNANPYMVARNTVHVLPREMFGFSTFGIAMALLKWLPIKV
VDKLVLAAARLMLGDTARYGVRRPKTGPIELKLVTGKTPVLDVGQVAQIRSGNIKVMEGV
KEITRNGAKFMDGQEKEFSAIILATGYKSNVPTWLKSCESFTKDGMPKTPFPMGWKGE
NGLYTVGFTRRGLLGTASDAVKIAKDIADQWMTVKDKSYRNSHIILLKST*

(6) GmYUCCA6(GLYMA_04G079700)
MEYFLREIEGKQAHDPLFMNNNKSSLSSSSSSSFSTVWVHGPVIVGAGPSGLAAAACL
REKSVPSVILERSNCIASLWQLKTYDRLRLHLPKQFCELPFMGFPSHFPTYPSKQQFVQ
YLENYAERFGIRPRFNETVQHAEFDAKLGLWRVKSVDKAEKTTEYVCRWLIVATGENAE
AVVPDIEGVEEFGAPIKHTSLYKSGEEFRGKRVLVVGCGNSGMEVCLDLCNHNATPSLV
VRDTVHVLPREMLGKSTFGLSMWLLKWLPIRLVDRFLLMVSWLLLGDTSKLGLDRPRLG
PLELKNLSGKTPVLDVGTLAKIKGGDIKVRPGIKRLKRQTVEFVDGRTENFDAIILATGYK
SNVPYWLKEEDMFSKKDGYPRRPFPNGWKGRNGLYAVGFTKKGLLGASMDAKRIAEDI
EQSWKAGANHRTTFARSHLPQPNS*

(7) GmYUCCA7(GLYMA_05G231100)
MLYMEYLKEVEGKTVHDYLEVKMGKMTKPIGVEGPVIVGAGPSGLAAAACLKQKGIPSLI
LERDDCLASMWQLKTYDRLCLHLPKQFCQLPLMPFPQNFPSYPTKQQFLAYLKAYADH
FDIKPALSKTVISANFDHRCGYWRVKTQGVKKEETEYVCQWLIVATGENAEEVVPQIEG
MSEFEGPILHTSSYKSGSMFCGKNVLVVGCGNSGMEVCLDLCNHHARPSLVVRDTVHIL
PQQMFGKSTFGLSLSLLNWFPMRLVDKFLLLMSHLILGDTAQFGLNRPKIGPLELKNLCG
KTPVLDFGTLAHIKSGKIKVCRGIKQLAQHKAEFVDGKIEDFDVIILATGYKSNVPTWLKG
SDMFCEKDGLPRKPFPNGWRGENGLYAVGFTKRGLLGASFDAKRIAGDIEHCWKAAN*

(8) GmYUCCA8(GLYMA_06G072100)
MESLKAQEGGEEQVKCVWVHGPIIVGAGPSGLAVAACLSHHGVPYVILERTNCITSLWQ
HRTYDRLKLHLPKHFCELPLIPFPLHFPKYPSNSYASRFNIRPRFNQSVQTAQFDPCSQL
WVVKTNGFQYISPWLVVATGENAEPVVPSISGMDKFRGPIVHTSVYKSGSDYKNQRVLV
IGCGNSGMEVSLDLCRHNANPYMVARNTVHVLPMEMFGFSTFGIAMALLKWLPIKLVDK
LVLAAARLMLGDTARYGVRRPKTGPIELKLVTGKTPVLDVGQVAQIRSGNIKVMEGVKEIT
RNGAKFMDGQEKEFSAIILATGYKSNVPTWLKSCESFTKDGMPKTPFPMGWKGENGLY
TVGFTRRGLLGTASDAVKIAKDIADQWMTVKDRSYCNSHIIFLKST*

(9) GmYUCCA9(GLYMA_06G081300)
MEYFLREIEGKQAHDPLFMNNYNKSSSSSSSFSSSSSSTVWVHGPVIVGAGPSGLAAA
ACLRDKSVPSVILERSNCIASPWQLKTYDRLRLHLPKQFCELPFMGFPSHFPNYPSKQQ
FVQYLENYAESFGIRPRFNETVQHAEFDGKLGLWRVKSVDKVGKTTEYMCRWLIVATGE
NAEAVVPDIEGVEEFGAPIKHTSLYKSGEEFRGKRVLVVGCGNSGMEVCLDLCNHNATP

SLVVRDTVHVLPREMLGKSTFGLSMWLLKWLPMRLVDRFLLMVSWLLLGDTSHLGLDR
PRLGPLELKNLSGKTPVLDVGTLAKIKGGDIKVRPAIKRLKRHTVEFVDGRTENYDAIILAT
GYKSNVPYWLKEEDMFSKKDGYPRRPFPNGRKGRNGLYAVGFTKKGLLGASMDAKRIA
EDIEQCWETGANHRTTLAPSHLPQPNS*

(10) GmYUCCA10(GLYMA_06G120700)
MEIEVPVVIVGAGPAGLATAACLNKYSIPNVVLERDDCHASLWRKRAYDRLKLHLGKDFC
NLPHMPFPPDFPTFVPRVDFLRYLDNYVTRFKISIRYNRNVESASMDEQNNGKWRVVVK
DTTTNADEVYVANYLVVATGENSEGYVPQIEGLEGFEGKHMHCSEYLNGRDLYGKHVLV
VGCGNSGMEIAYDLSNWGANTSIVVRGPVHYFTKEMVYVGMSLLKYFKIEKVDKLMLLM
SKLKYGDMSNYGLIRPKDGPFFLKKKGGTTPTIDVGCVSRIKKGEVKVFPAISSIKEDKLIE
FEDGQNGQFDVIIFATGYNSTVLKWLKDYRGLFNENGMPKPDFPNHWKGENGIYCAGF
SRRGLDGIAFDAKRIAADIKKTVNIKNLPSEEDNFASFKLLEK*

(11) GmYUCCA11(GLYMA_06G152700)
MEEVSVIIAGAGPSGLAISACLSQNSISHIILEKDDCSASLWRKNAYDRLKLHLASEFCALP
LMPHSPSSPTFLSKDEFVQYIDSYIARFNINPLYCRFIEFAAYDEVENKWRVEAKNTLEGT
REIYVAKFLVIATGENSEGYIPDVHGLESFEGEIMHSKYYKSGSKYESKEVLVVGCGNSG
MEIAYDLNDWGANTSILIRNPVHVFTKELINEGMRMMKHLPIHVVDTIITSLANMEYGDLS
KYGIYQPKKGPFQLKFITGRAPVIDVGTIRRIKEGAIKVIPSHIVRIENKKVIFGNDVEKKFD
VIVFATGYISVANKWLKDYKYILNDEGMPKNDFPNHWKGDRGLYCAGLSNRGLFGVKM
DAEAIADDINQTLKLH*

(12) GmYUCCA12(GLYMA_07G086200)
MDYLKELEGKSVHDCYHQHQIKMSKMASPIFVPGPVIVGAGPSGLAAAACLKQKGIPSL
VLERAQCLASMWQFKTYDRLRLHLPKQFCQLPLMPFPKNLPSYPTKQQFLAYLKAYADH
FDIKPVFSQTVVSAEFDHVCHHWRVKTQGVLKKEDTAEYVCQWLIVATGECAEEVVPQI
EGMGEFEGQIVHTCKYKSGNKFCGKNVLVVGCGNSGMEVCLDLCNHNARPSLVVRDT
VHILPQQMLGKSTFGLSMFLLKWFPIRFVDQFLLLMSHLMLGDTDQFGLRRPKLGPLEL
KNLYGKTPVLDVGTLTQIKNGKIKVCRGIKRLARNAVEFVDGKVENFDAIILATGYKSNVP
SWLKGSDMFSEKDGFPRKPFPNGWKGENGLYAVGFTKRGLLGASIDAKRIAEDIEHSW
KAEATHVLEFPCPLA*

(13) GmYUCCA13(GLYMA_08G038600)
MLNMEYLKEVEGKSVHDYVEVKMGKMTKPISVAGPVIVGAGPSGLAAAACLKQKGIPSLI
LERADCLASMWQLKTYDRLCLHLPKQFCQLPLMPFPQNFPSYPTKQQFLAYLKAYADHF
DIKPALSKTVISANFDHGCGYWRVKTQGLKKEETEYVCQWLIVATGENAEEVVPQIEGM
SEFEGPILHTSSYKSGSMFGGKNVLVVGCGNSGMEVCLDLCNHDARPSLVVRDTVHILP
QQMFGKSTFGLSMSLLKWFPMRLVDKFLLLMSHLILGDTAQFGLNRPKIGPLELKNLCG
KTPVLDVGTLAHIKSGKIKVCRGIKQLAKHKVEFVDGKTENFDVIIMATGYKSNVPTWLKG
SNMFCEKDGLPRKDFPNGWKGENGLYAVGFSKRGLLGASIHSKRTAEDIEHCWKAANT
TRV*

(14) GmYUCCA14(GLYMA_09G190700)

MDYLKELEGKSVHDCYHHHQQQQIKMSKMASPIFVPGPVIVGAGPSGLAAAACLKQKGI
IPSLILERAQCLASMWQFKTYDRLRLHLPKQFCQLPLMPFPKNLPSYPTKQQFLAYLKAY
ADHFDIKPVFSQTVVSAEFDHVCQLWRVKTRGVIKKEDTAEYVCQWLIVATGECAEEVV
PQIEGMGEFEGQIVHTSKYKSGSMFCGKNVLVVGCGNSGMEVCLDLCNHNARPSLVVR
DTVHILPQQMLGKSTFGLSMFLLKWFPIRFVDQFLLLMSHLMLGDTAQFGLRRPKLGPL
ELKNLYGKTPVLDVGTLTQIKNGKIKVCRGIKRLARNAVEFVDGKVENFDAMVLATGYKS
NVPSWLKGSDMFSEKDGFPRKPFPNGWKGENGLYAVGFTKRGLLGASIDAKRIAEDIEH
SWKAEAKHVLEFPRPLA*

(15) GmYUCCA15(GLYMA_10G128700)
MHDICLNMPPMVQTFDPEDLFKRRCIWVNGPVIVGAGPSGLAVAACLKEQGVPFLILER
ANCIASLWQNRTYDRLKLHLPKQFCQLPNFPFPEDFPEYPTKFQFISYLESYAKHFNISP
QFNETVQSAKYDETFGLWRVKTIRKIKKLGEASSGCCGAVECEVEYICRWLVVATGENS
EKVVPEFEGLGEFGGHVMHACDYKSGEGYGGQKVLVVGCGNSGMEVSLDLCNHNAN
PSMVVRSSVHVLPREAFGKSTFELAVMLMKRFPLWMVDKILLVLARLILGNVEKYGLKRP
SVGPLELKHTAGKTPVLDIGALEKIRSGKIKVVPGIRRFFPGKVELVDGQVLQIDSVVLAT
GYHSNVPSWLKENDFFTSDGTPRNPFPNGWRGKGGLYAVGFTRKGLSGASLDAINVAH
DIAKNWKEENKQKRKTVAARHRRCISHF*

(16) GmYUCCA16(GLYMA_14G128200)
MGRSCNKSQQQHVEGPIIIGAGPSGLAVAACLSEDKVPFVILERHNCIASLWQNKTYDRL
KLHLPKQFCELPLKGFPHTFPKYPTKYQFISYMESYASHFNIHPIFNQTVKSAEFDKGSN
VWVVRTEEFEYSSRWLVVATGENAEPVVPRIHGMELFGGAVAHTSVYKSGSEYRNKKV
LVIGCGNSGMEVCLDLCRHNAKPYMVARNTVHVLPREMLGFSTFGIAMALYKWFPIKLV
DKIILLATNLILGNTNHYGIKRPKTGPIELKLATGKTPVLDVMEGVKEITRNGAKFMDGKEK
EFDAIILATGYKSNVPTWLKGCDFFTKDGMPKTPFPHGWKGEQGMYTVGFTRRGLHGT
SCDAIKIAEDIAEQWRTVEDKSHCDSHIILLNNS*

(17) GmYUCCA17(GLYMA_14G141200)
MDYCLRELEGKQAHDSLFLEKMKNSLSQRTTTSERCVWVPGPVIVGAGPSGLATAAYLK
EKGVPSLILERSNCIASLWQLKTYDRLHLHLPKNFCELPLMGFPCDFPTYPTKQQFIEYLE
SYAERFHIRPRFNETVQHAEFDATLGFWRVKSLNKREVATEFVCRWLIVATGENAEAVVP
GIEGMGEFGGTIKHTSLYKSGEEFRGKRVLVVGCGNSGMEVCLDLCNHNATPSLVVRDT
VHILPREMLGKSTFGLSMWLLKWLPIRFVDRFLLIVSWLMLGDTARFGLDRPKLGPLQLK
NLSGKTPVLDVGTLAKIKSGHIKVRPGIKRLKRYTVEFVDGRTENFDALILATGYKSNVPY
WLKEEDMFSKEDGFPTKPFPNGWKGENGLYAVGFTKRGLLGASMDAKRIAEDIERCWK
AKHSTSFSLSLNVPQSNS*

(18) GmYUCCA18(GLYMA_17G189700)
MDYCLRELEGKQAHDPLFLQKMKNSSSQSTTTSSERCVWVPGPVIVGAGPSGLATAAY
LKEKGLPSLILERSNCIASLWQLKTYDRLHLHLPKNFCQLPLMGFPCDFPTYPTKQQFIEY
LESYAESFDIRPRFNETVRRAEFDATLGFWRVKSFNKKEVATEFVCRWLIVATGENAEAE
VPEIEGMGEFGGAIKHTSFYKSGEEFRGKRVLVVGCGNSGMEVCLDLCNHNATPSLVV
RDTVHILPREMLGKSTFGLSMWLLKWLPIRFVDWFLLIVSWLMLGDTARFGLDRPKLGP

LQLKNLSGKTPVLDVGTLAKIKSGHIKVRPGIKRLKRYTVEFVGGRTENFDAIILATGYKS
NVPYWLKEDDMFSKEDGFPTKPFPDGWKGENGLYAVGFTKRGLLGASMDAERIGEDIE
RCWKEKHSSTSFSLSLNVPQSNS*

(19) GmYUCCA19(GLYMA_17G205800)
MGSCNKPQQQQQQHVQGPIIIGAGPSGLAVAACLSEHKVPFVILERHNCIASLWQNKTY
DRLKLHLPKQFCELPLKGFPHTFPKYPTKYQFISYMESYASHFNIHPIFNQTVESADFDK
GSKVWVVKTQEVDYSSRWLVVATGENAEPVVPRIHGMELFNGDVAHTSVYKSGSEYRN
KKVLVIGCGNSGMEVCLDLCRHNAKPYMVARNTVHVLPREMFGFSTFGVAMALYKWFPI
KLVDKIILLATNFILGNTNHYGIKRPKTGPIELKLATGKTPVLDVGQVAQIKCGNIKVMEGVK
EITRNGAKFMDGQEKEFDAIILATGYKSNVPTWLKGCDFFTEDGMPKTPFPHGWKGEQ
GLYTVGFTRRGLQGTSCDAIKIAEDIAEQWRTVEDKSHCDSHIILLNNS*

(20) GmYUCCA20(GLYMA_19G170800)
MENLLRLVDHEDFFSRRCIWVNGPIIIGAGPSGLATAACLREQGVPFMVLERADCIASLW
QKRTYDRLKLHLPKQFCQLPKLPFPEDFPEYPTKKQFIEYLESYAKHFEINPQFNECVQS
ARYDETSGLWRVKTVSSSGAAARGEIEYICRWLVVATGENAECVMPDIEGLSEFKGDVIH
ACDYKSGESFRGKKVLVVGCGNSGMELSLDLCNHHASPSMVVRSSVHVLPREVFGIST
FELAVMLLQWLPLWLVDKILLILAWFVLGNIEKLGLKRPSMGPLELKNTKGKTPVLDIGAL
EKIRSGDIEVVPGIKRFNNGEVEFVNGEKLDIDAIVLATGYRSNVPSWLQEGEFFSKNGY
PKMPFPHSWKGNAGLYAVGFTKRGLSGASSDAVKIAQDIGQVWKNETKQKKQRTTACH
RRCISQF*

(21) GmYUCCA21(GLYMA_19G206200)
MQEATKVIIIGAGTSGIATAGCLTKQSIPYIMLEREDCFASLWQKYTYDRLHLHLRKQVCEL
PHLPFPKSYPHYVPRKQFIDYLGNYVNHFEIKPLYQRAVELVEYDGWKGIWRVKAQNRR
SGELEEYAGKYLVVASGETAEPRLPQIQGLESFNGKVIHSTAYKNGNEFKNKHVLVVGSG
NSGMEIALDLSNFGAKPSIIVRSPVHFLSRDMMYYASLMLNYLSLSTVEKVLVMVSKVVY
GDLSEYGIPYPSEGPFTMKMKYAKFPIIDVGTVKKIKSREIQVLPAEIKSIRGNEVLFQDGK
SYTFDSIVFCTGFKRSTQKWLKGGDDLLNEDGFPKNSFPNHWKGRNGLYCVGLSRRGF
FGANMDAQLVANDIASLIPQEEREGICVM*

(22) GmYUCCA22(GLYMA_20G080000)
MHDICLNMPPMVQTFDPEDLFTRRCIWVNGPVIVGAGPSGLAVAACLKDQGVPFIILERA
NCIASLWQNRTYDRLKLHLPKQFCQLPNVPFPEDFPEYPTKFQFISYLESYAKHFSIAPQ
FNETVQSAKYDETFGLWRIKTIRKIKKLGGLSSGGCAECEVEYICRSLVVATGENSEKVV
PEFEGLGEFGGHVMHACDYKSGEGYGGQKVLVVGCGNSGMEVSLDLCNHNANPSLVV
RSSVHVLPREVFGKSTFELAVTLMKRFPLWIVDKILLILARLILGNVEKYGLKRPSVGPLEL
KHTAGKTPVLDIGALEKIRSGKIKVVPGIRRFLPGKVELVDGQVLQIDSVVLATGYHSNVP
SWLKEDDFFTNDGTPRNPFPNGWRGKGGLYAVGFTRRGLSGASLDAINVAHDIVKSWK
EETKQKRKTVAARHRRCISHF*

(23) GmYUCCA23(GLYMA_10G041800)
MENLFRLVDYEDMFSKRCIWVNGPVIVGAGPSGLATAACLKQQGVPFMVLERAECIASL

WQKRAYDRLKLHLPKQFCQLPNLPFPKDFPEYPTKKHFIDYLESYAQKFEINPRFNECV
QCARYDETSGLWRVKTVATCGAAKSEFEYICRWLVVATGENAECVIPEIEGLGEFKGDVI
HACEYKSGESFKGKKVVVVGCGNSGMELSLDLCNHNASPSMVVRSSVHVLPREVFGK
STFELAVLMLQWVPLWLVDKILLVLAWLVLGNMERFGLKRPSEGPLLLKNTKGKTPVLDI
GTLEKIRSGDIKVVPEIKRFTNGCVEFVNGEKQHVDAVVLATGYRSNVPSWLQEGEFFS
KNGFPKSPFPNGWKGNGGLYAVGFTRRGLSGASSDAMKIAQDIGQVWKQETRQKKQR
TNACHRRCISQF*

(24) GmYUCCA24(GLYMA_13G128800)
MENLFRLVDYEDMFSKRCIWVNGPVIVGAGPSGLATAACLKQQGVPFMVLERAECIASL
WQKRTYDRLKLHLPKQFCQLPNLPFPKDFPEYPTKKHFIDYLESYAQKFEINPRFNECV
QCARYDETSGLWRVKTVATCGSAKSEFEYICRWLVVATGENAECVIPDIEGLGEFKGDVI
HACEYKSGESFKGKKVVVVGCGNSGMELSLDLCNHNASPSMVVRSSVHVLPREVFGK
STFELAVLMLQWLPLWLVDKILLVLTWLVLGNMERFGLKRPSEGPLLLKNTKGKTPVLDIG
TLEKIRSGDIKVVPEIKRFSNGYVEFVNGEKQGVDAVVLATGYRSNVPSWLQEGEFFSK
NGFPKSPFPNGWKGNAGLYAVGFTRRGLSGASSDAMNIAQDIDQVWKQETRQKKQRT
TACHRRCISQF*

4. OsYUCCAs

(1) OsYUCCA1(Os01g0645400)
MDNKPAQERRETWVPGAVIVGAGPSGLAAAACLAARGVPATVLERSDSLASTWRHRMY
DRLALHLPKRFCELPLLPFPEEYPTYPSKDQFVAYMEAYAAAAGVAPRFGATVEEAAFDA
AVGAWRVRLDGGEVLMARWLVVATGENAEPRVPDFPGMQKFAGCAMHTSEYKSGEQF
AGKKVLVVGCGNSGMEVSLDLCRHGAKPSMVVRNTVHVLPREMFGLSTFGIAMALLRW
LPVQLVDRFLLTAAHLILGNTGQFGLRRPKTGPIELKNLTGRTPVLDVGTLDHIKSGKIKVV
GAVKEMTRQGVRFTDGKEEQFDTIILATGYRSNVPSWLKDAGDLFTREGISKVPFPNSW
RGRNGLYTVGFTQRGLLGTSSDALNVAKDIHCQWRERDRSAINVLEISNSSF*

(2) OsYUCCA2(Os05g0528600)
MLVWVQGPIVVGAGPSGLAAAACLKEKGIDSLVLERSSCLAPLWQLKMYDRLSLHLPRQ
FCELPLFPFPASYPDYPTKQQFVAYLESYAAKFGINPMYNHTVVCAEFDERLMLWRVRT
TQATGMMEDDVEYVSQWLVVATGENSEAVLPVIDGLEEFRGSVIHTSAYKSGSKFAGKT
VLVVGCGNSGMEVCLDLCNHNGYPRIVVHILPREMLGQPTFRLAMWLLKWLPIHIVDRIL
LLVARAILGDTSQFGLKRPSLGPLELKSLSGKTPILDIGTLAKIKSGDIKVRPAIRRIAGQQV
KFVDGRSEQFDAIVLATGYKSNVPCWLKVYGNTADVPFAVEEFQYFFS*

(3) OsYUCCA3(Os01g0732700)
MDPWSEIEGKRAHDPIFQNYFSQNCRQSVDGFCKKRSADAAVARAERCIRVLGPIIVGA
GPSGLAVAACLKEKGVDSLVLERSNCIASLWQLKTYDRLSLHLPRQFCELPLMPFPAYYP
IYPSKQQFVAYLESYAARFGICPTYNRTVVCAEYDEQLQLWRVRTRATGIMGEEVEYVSR
WLVVATGENAEVVLPEIDGLDDFKGTVMHTSSYKSGGAFAGKRVLVVGSGNSGMEVCL
DLCNHNANPHIVVRDAVHILPREMLGQSTFGLSMWLLKWLPVHVVDRILLLIAQTMLGDT
AQLGLKRPTIGPLELKSLSGKTPVLDVGTFAKIKSGDIKVRPAIKQISGRQVEFMDTRLEE
FDVIVLATGYKSNVPFWLKDRELFSEKDGLPRKAFPNGWKGENGLYSVGFTRRGLMGT

SVDARRIAHDIEQQWKARGKHPGVLL*

(4) OsYUCCA4(Os01g0224700)
MDCFAETEGKRAHDPLYQRRAAAAATPATGVPVDDVDKVVDVPGAVIVGAGPAGVAVG
ALLGLRGVAYVVLERCGCIASLWRHRTYDRLCLHLPKRFCELPLRPFPASFPEYPTRDQF
LGYLDAYAREFGVEPVFRRAVISAEYDGESWWVYTREVVAAAAGGEQAVLGCTMTVYR
SRWLVVATGENAEPVVPEMDGAGRFKGQMMHSSEYRNGDGYAGKKVLVVGCGNSGM
EVSLDLCNHNARASMVVRDTVHVLPREILGFSTFGLSMWLLRWLSVQTVDWLVLLLSFL
VFGDTARLGIPRPSLGPFELKSVSGKTPVLDVGTLAKIKSGDIKVTPAIQCFQEHGVEFVD
GSTEEFDVVILATGYKSNVPYWLKEKEFFSEKDGFPRKGNAWKGQNGLYAVGFSRRGL
SGVSMDANNIVQDIVQRLHDMGYERSENN*

(5) OsYUCCA5(Os12g0512000)
MPTSQQDGGGGGERLFHGGGAGDGDGGGGGGAAAAAAARQVWVPGPVIVGAGPSG
LATAACLKARGVPSLVLDKDATVAASWRERTYERLRLHLPRGFCELPLAPPFPPGTPPYP
TRDQFVAYLDAYARAFAVEPRLGSRVRAASYDAAIGFWRVAAVDEAGGGGAGGETEFLS
RWLVVATGENAVAAWPAEGVGAYRGAVMHTSSYKRGDEFAGKKVLVVGCGNSGMEVS
LDLCNNGAATSMVVRDKIHVLPREILGISTFGLSVFLLKWFPIKWVDALLLIFSRLILGNIEK
YGLRRPKIGPLQIKCSTGKTPVLDIGALKKIKNGEIKVVPAIHCFTEDGVEFVNGCREDFD
AVIFATGYKSNVPSWLKEEEFFSESDGFPRKAFPHSWRGKNGLYATGFTKRGLQGTSYD
AAMIAADIARRWTKSLAGPTAAADADHHETYIAN*

(6) OsYUCCA6(Os07g0437000)
MAARVVWVNGPIVVGAGPAGLSVAACLRERGVPSVLLERADCIASLWQRRTYDRLRLHL
PKHFCELPGMPFPDGYPEYPDRRQFVDYLQAYAARAGVEPRFNQSVTSARYDDAAGL
WRVRAEDVSVDAAGDVTEYIGRWLVVATGENAERVVPEIDGADDFEGPVSHVAEYKSG
AAYRGKRVLVVGCGNSGMEVCLDLCHHNALPAMVVRDSKVHVLPREMLGVATFSVAVF
LLRFLPLWVVDRILVVLAWLFLGDLAKIGITRPSRGPLELKNTRGRTPVLDIGALARIRSGD
IEVVPGIRRLLRGGAELVDGRRVPADAVILATGYQSNVPQWLKGSDFFTQEGYPRVPFP
DGWKGESGLYSVGFTRRGLSGVSSDAVKVAQDIAMAWNHQTATTR*

(7) OsYUCCA7(Os04g0128900)
MVLQHSDRMDSLFSPQTSWVSGPIIVGAGPSGLAVAASLREQGVPFTMLERADCIASLW
QKRTYDRLKLHLPKQFCELPRMAFPAHYPEYPTRRQFIDYLEDYAAAFDINPLFGHTVLS
ARYDETSGLWRVRASSSAGAEMEYIGSWLVVATGENAESVVPDIPGIDGFGGEVVHVAD
YKSGEAYRGKRVLVVGCGNSGMEVSLDLCDHGARPAMVVRDAVHVLPREVLGKSTFEL
AVLLMAWLPLWLVDKILVLLAWLVLGNLAKLGIRRPATGPLELKNTTGRTPVLDYGALARI
RSGEITVVPGVARFGRGFAELADGRVIALDAVVLATGYRSNVPQWLQGNDFFNKDGYPK
TAFPNGWKGESGLYAVGFTRRGLSGASADAMRAAKDLARVWKEATKPTKKSTACHRRC
ISVIF*

(8) OsYUCCA8(Os03g0162000)
MQGQQKQNAGGGGGDNASPCIVLDGPIIVGAGPSGLAVAATLRQHGAPFTVVERSGGV
ADLWTNRTYDRLRLHLPKVFCELPHVAFPPDFPTYPTKHDFLRYLHSYAARFAIAPLLRR

TVTRAWYDHPASLWRVTTTTTSSSATSVITEYASPWLVVASGENAEVVVPKVKGRERFA
GEALHSSEYRSGERFRGMRVLVVGCGNSGMEMCLDLCEHGAMPFMSVRSGVHVLPR
EMFGASTFGIAMKLLRWLPIKMVDRFLLLVARMVLGDTEKYGLKRPKLGPLEIKNITGKSP
VLDVGAWSLIKSGNIKIVPEVESFSGNGARFVDGNEMAFDAVIFATGYRSNVPSWLQED
GELFTEEGKLRSSGSSSEWRWRGPNGLYCVGFSGRGLLGAGADALRAAADIAGRWQE
TQQAAANISSV*

(9) OsYUCCA9(Os01g0273800)
MAAVEQDQEEEVIIVGAGPSGLAAAACLSVRGVTGCLVLERDDCVASLWRHRTYDRVRL
HLAKRYCALPHAPHGEASPTYLPRDDFLRYLDAYASRFGVRARLRREVRSARYDAARA
RWLVDAVDLATGRAERYAARHLVAAAGENDERVVPEVPGMETFPGKVVHAADYRSAEG
FKGKSVLVVGGGNSGMEIAYDLAVGGAATSIVIRSELHLVSKEIWNLAMTLYRYLPVWVID
KVVLLMCAAVFGDTARYGLRRPAVGPFTMKATTTMYPVVDVGTFAKIRSGEIRVLPAAIK
GVRGRDVEFADGQRHAFDAVVFATGYRSTTKHWLKSDDGLIGDDGMAGRSYPDHWKG
ENGLYCAGMVRRGIYGSYEDAEHIADDISKQLRSSSKPTHNNGSA*

(10) OsYUCCA10(Os01g0274100)
MAVAAEGEEMTPEHEEEVIIVGAGQSGLAAAACLSVRGVASCLVLERDDCVASLWRHRA
YDRLRLHLPKRHCALPRAPHAAAAPDYLPRDDFAAYLDAYASRFGVRTRLRREVRSARH
DAARARWLVEAVDLATGKAERYAARHLVAAAGENDERVVPEVPGMDTFPGKVVHSADY
RSAGAFKGRSVLVVGCGNSGFEIAYDLAAGGAAAVSIAVRGEVHLVSREVWSVGMALQ
RYLPTWAVDKVVLLMCAVVFGGDTARYGLRRPAVGPFSMKMTTPAYPVFDVGTFAKIRS
GEIRVVPAGIKSVRGGDVEFADGRRHAFDAIVFATGYRSTTKQWLKSDDGLIGDDGMAG
RSYPDHWKGENGLYCAGMVRRGIYGSGEDAELIADDISKQMKRWSSEPVYNGHISNGS
P*

(11) OsYUCCA11(Os12g0189500)
MCSPRIYTSHQEKKQHIQNSVPTLVSLPSLSFYFLMEKALVLIVGAGPSGLATAACLGQLS
IPYVIIEREDCTASLWRKHTYDRLKLHLAKEFCEMPHMPYPEDTPTYIPKIQFLRYMDDYV
EHFNICPKFNSSVESCLYDDVQKYWVVTTHDQVNGMVSKYAARFLVVASGENSAGNIPS
IPGLEDFSGHVIHSSSFRSADSYAAQRVLVVGCGNSGMEIAYDLSSHGANTSIVIRSPLHV
MTKELIHMGMKLASWSLPVKFVDFILVVLAYLWFGNLSKYGIVRPNKGPLLLKANTGRSA
VIDVGTVELIKKGDIKVFGTISCIKGNVVEFDDGKESYFDAIVFATGYTSTANNWLKNGED
MMNKEGMPKKDFPNHWKGSNGLYCVGFARRGLSGIAHDAKNVANDVKAFLDLMAPF*

(12) OsYUCCA12(Os02g0272200)
MEKALVLIVGAGPSGLATAACLGQLSIPYVIIEREDCTASLWRKHTYDRLKLHLAKEFCEM
PHMPYPEDTPTYIPKIQFLRYMDDYVEHFNICPKFNSSVESCLYDDVQKYWVVTTHDQV
NGMVSKYAARFLVVASGENSAGNIPSIPGLEDFSGHVIHSSSFRSADSYAAQRVLVVGC
GNSGMEIAYDLSSHGANTSIVIRSPLHVMTKELIHMGMKLASWSLPVKFVDFILVVLAYLW
FGNLSKYGIVRPNKGPLLLKANTGRSAVIDVGTVELIKKGDIKVFGTISCIKGNVVEFDDG
KESYFDAIVFATGYTSTANNWLKNGEDMMNKEGMPKKDFPNHWKGSNGLYCVGFARR
GLSGIAHDAKNVANDVKAFLDLMAPF*

(13) OsYUCCA13(Os11g0207700)
MEAVEEVVVLIVGAGPAGLATAACLAQRHVPYVIVERESCTASLWRHRAYDRLKLHLAKE
FCELPHMAYPVGTPTYIPRDMFVEYLDSYTDQFGIRPRYHTAIESAIYDGGKNRWAVLAR
DTDTSVVTRLTAQFLVVATGENSAASIPPVPGLTRFEGEAIHSSAYKSGRAYTGKNVLVVG
AGNSGMEIAYDLATHGAHTSIVVRSPIHIMTKELIRFGMTVVQNLGLTVTTADSLLVMAAN
FYFGDLSKHGITRPKIGPLLLKSQTGRSAVIDVGTARLIKGGVIKVFQGISKIKTNSIEFHGG
KQIPFDAIVFATGYKSTVNTWLKNGESMFKDDGFPKKFFPNHWKGENGLYCAGFARRG
LAGIAMDAKNIADHIVATMDQVSC*

(14) OsYUCCA14(Os11g0207900)
MNAMEDVVVLIVGAGPAGLATAACLAQRHVPYIIVERESSTASLWRHRAYDRLKLHLAKE
FCELPHMAYPAGTPTYVPRDMFVEYLDSYANQFGIRPRYHTAVESAIHDKGKNQWVVLV
RDMDTSVVARLATQFLVVAAGENSAANIPPIPGLSRFEGEAIHSSAYKSGRAYTGKSVLV
VGAGNSGMEIAYDLATHGAHTSIVVRSPVHIMTKELIWYGMTMVQNLGLNVTAVDSLLVM
AANFYFGDLSKHGIMRPKMGPLLLKSQTGRSAVIDVGTARLIKGGVIKVFQGISKINTNSV
EFHGGRQNSFDAIVFATGYKSTVNAWLKNGESMFKDDGFPKNYFPNHWRGENGLYCA
GFARRGLAGIAMDAKNIANDIVAAMDKMSC*

5. MtYUCCAs

(1) MtYUCCA1(Medtr1g008380)
MDCYLRELEGKQAHDPLFIEKMINNNKNSTSSSDRCLWIPGPLIVGAGPSGLAVAAYLKQ
KGVPSLILERSNCIASLWKLKTYDRLRLHLPKQVCELPLMEFPSGFPTYPTKQQFIEYLES
YSKNFDIRPWFNETVMHAEFDATLGFWRVRSEGKAGMVTEFVCRWLIVATGENAEAVV
PEIEGVDEFVGSIRHTSLYKSGEEFRGKKVLVVGCGNSGMEVCLDLCNHDAAPSIVVRD
SVHILPRDMLGKSTFGLSMWLLKWLPVQLVDHILLTVSWLMLGNTERFGLVRPRLGPLE
LKKLSGKTPVLDVGALAKIKRGDIKVRPGIKRLKRYTVEFADGSTENFDAIILATGYKSNVP
YWLKDKGMFSKEDGYPRKPFPNGWKGENGLYAVGFTKRGLLGASMDAKNIAEDIERC
WKAEAKHIFPQSNS*

(2) MtYUCCA2(Medtr1g011630)
MDPFKEKVKCVWIHGPIIVGAGPSGIAVAACLSEQGVPSLILERSDCIASLWQNRTYDRLK
LHLPKHFCELPMMSFPQTFPKYPTKHQFISYMESYADHFHIHPRFNQTVLSAEFDSTSQI
WMVRTKEGDFQYFSPWLIVATGENAEPVFPTIHGMEHFHGPVVHTSDYKSGSEYKNKK
VLVIGCGNSGMEVSLDLCRHNAMPHLVARNSVHILPRDMFGFSTYGIAMGLYKWLPLKL
VDKFLLLVSSFFLGNTNHYGIKRPKTGPIELKLATGKTPVLDVGQIAQIKSGNIKVMEGVK
EITRNGAKFLDGQEKEFDAIILATGYKSNVPSWLKGNDFFTKDGMPKTPFPHGWKGEQ
GLYTVGFTRRGLHGTYFDAIKIAEDITNQWKTLKSKSCCDSHIILLNNS*

(3) MtYUCCA3(Medtr1g046230)
MPPMSQTFDPEDILKNRCILVNGPVIVGAGPSGLAVAACLKDQNVPFVVLERANCIASLW
QNRTYDRLKLHLPKQLCQLPNFPFPDHFPEYPSKFQFISYLESYAKNFNITPNFNETVLS
AKYDETFGLWRIKSNRIKSDKVVEVEYICRWLVVATGENSEKVVPEFEGLSDFGGNVMH
ACDYKSGVNYSGKNVLVVGCGNSGMEVSLDLCNHNANPSMVVRSSVHVLPREIFGKST
YEVAVMLMKRLPIWMVDKILLGLTRVILGNVEKYGLKRPCMGPLELKNTSGKTPVLDIGAL

EKVRSGEIKVVSGIKRFSQGKVELVDGKVLDIDSVVLATGYRSNVPSWLKENEFFSKDG
MPKDPFPNGWKGNAGLYAVGFTRRGLSGASLDAISVSQDIAKSWKEENKQRKKTVAAR
HRRCISHF*

(4) MtYUCCA4(Medtr1g069275)
MENLIRLVDNEEDAVSHRCIWVNGPIIVGAGPSGLATAACLKQQGVPFMVLERSNCIASL
WQKRTYDRLKLHLPKQFCQLPNFPFPQEFPEYPTKKQFINYLESYAKHFEINPRFNECV
QSAKYDETNGLWRVETVSNGNEVEYICRSLVVATGENAECVVPYIEGLGGEFKGEIIHAC
DYKSGESFRGKKVLVVGCGNSGMELSLDLCNHNASPSIVLRSSVHVLPREVFGKSTFGL
AVMMMKWLPVWIVDKILLILAWLVFGNIEKYGLKRPLEGPLELKNTKGKTPVLDIGTLEKI
RSGHIKVVPGIKRFTNGYVELVNGEKNQVDAVVLATGYRSNVPSWLKEGEFFSKNGFPK
SPFPNGWKGTVGLYAVGFTKRGLSGSSYDAKQIAQDIAQVWKQEIKQKKHCTNACNKR
CISQF*

(5) MtYUCCA5(Medtr3g088925)
MEVEIPVVIVGAGPGGLATSACLNKHSISNIILEKDDCHASLWRKRAYDRLKLHLGKDFCN
LPHMPFPSDFPTFVPRVDFLRYLDDYVTHMKICIRYNRYVNDASFDATNGKWRVCVHDT
TLNLDEIYVADYLVVATGENCDPYIPMINGLESFEGEYLHCSKYLNGRPWYDKNVLVVGS
GNSGMEIAYDVSTWGANTSMVIRSPVHYLTKEMVYIGMSLLKYISIEKIDKLMVFMSKMV
YGDMSKYGLVRPKDGPFAMKKKGGRTPTIDVGCVKQIKKGKVKVYPAISSIKKGKIIEFAD
GKSGQFDVIVFATGYRSSVQKWLKDYKELFNENGMPKARFPDHWKGENGIYCAGFSQ
NGLQGIHFDALKIANDISFTVNAMKHHDAEANAEIKLLDE*

(6) MtYUCCA6(Medtr3g088945)
MEVEIPVVIVGAGPGGLATSACLNKHSISNIILEKDDCHASLWRKRAYDRLKLHLGKDFCN
LPHMPFPSDFPTFVPRVDFLRYLDDYVTHMKICIRYNRYVNDASFDATNGKWRVCVHDT
TLNLDEIYVADYLVVATGENCDPYIPMINGLESFEGEYLHCSKYLNGRPWYDKNVLVVGS
GNFGMEIAYDVSTWGANTSMVIRSPVHYLTKEMVYIGMSLLKYISIEKIDKLMVFMSKMV
YGDMSKYGLVRPKDGPFAMKKKGGRTPTIDVGCVKQIKKGKVKVYPAISSIKKGKIIEFAD
GKSGQFDVIVFATGYRSSVQKWLKDYKELFNENGMPKARFPDHWKGENGIYCVGFSR
NGLQGIHFDALKITNDISFTVNAMKHHDADANA*

(7) MtYUCCA7(Medtr3g088955)
MEVEIPVVIVGAGPGGLATSACLNKHSISNIILEKDDCHASLWRKRAYDRLKLHLGKDFCN
LPHMPFPSDFPTFVPRVDFLRYLDDYVTQMKICIQYNRYVNDASFDVTDGKWRVCVHD
TTLNVNEIYVADYLIVATGENCDPYIPMINGLESFEGEYLHCSKYLNGRLWYDKNVLVVGS
GNSGMEIAYDVSTWGANTSMVIRSPVHYLTKEMVYIGMSLLKYISIEKIDKLMVFMSKMV
NGDMSKYGLVRPKDGPFAMKKKGGRTPTIDVGCVKQIKKGKVKVYPAISSIKKGKIIEFAD
GKRGQFDVIVFATGYRSSVQKWLKDYKELFNENGMPKARFPDHWKGENGIYCAGFSQ
NGLQGIHFDALKIANDISFTVNAMKHHDAEANAEIKLLDE*

(8) MtYUCCA8(Medtr3g109520)
MDYCIRELEGKQANDPLFIKTMKNNKSSSTEGRVFVQGPVIVGAGPSGLAAAACLQQKN
IPCVILERSNCVASLWQLKTYDRLRLHLPKQFCELPFMEFPSNFPTYPSKQQFIKYLEDYA

GSFGIRPRFNETVQNAEFDGKIGCWRLKSFNSKADVTTEYVCRWLIVATGENAEAVVPNI
EGVDEFGGVIRHTSLYKSGEEFRGKKVLVVGCGNSGMEVCLDLCNHDATPSLVVRDSV
HVLPREMLGKSTFGLSMWLLKWFPLGLVDRFLLIVSWLMLGDTAQLGLDRPRLGPLQLK
NLSGKTPVLDVGTLAKIKGGHIKVRPSIKRLKRQTVEFVDGRSENFDGIILATGYKSNVPY
WLKEEDMFSKEDGFPMKPFPSGWKGKNGLYAVGFTKRGLQGASLDAKRIADDIELCLE
SEAKYGS*

(9) MtYUCCA9(Medtr4g051642)
MEIEMPVVIVGAGPGGLATSACLNKHSISNIILEKDDCHASLWRKRTYDRLKLHIGKKFCN
LPHLPIPSDLPNFVPRVDFLRYLDNYVNHFKICIRYNRYVDDASFDVKSGKWRVCVRDN
RLNIDEIYVADYLVVATGENCDEYIPMINGLESFEGEYLHSSKYLNGRSWYDKNVLIVGSG
NSGMEIAYDVSNWGANTSIVIRSHMHPLTKEMVFVGMCLMKYLSVEKVDKLLVFMSKM
MYGDMSKYGLIRPKEGPFSIRVRTGRPPSIDVGCIKMIKKGKVKVYPTISRIKKGKIVEFV
DGKSGQFDVIVFATGYRSNVLEWLKDYKELFNENGMPKGCYPNHWKGENGLYCAFSR
RALQGIVYSSQKIANDISLTIDAKKLLAEAVEVNARIKLLDE*

(10) MtYUCCA10(Medtr5g033260)
MQEFTVVIVGAGPSGLAISACLTQNSISHIILEKDDCCASLWRKNAYDRLNLHLASEFCSL
PLMPHPPSGPTYLSKYQFLQYIDKYVAHFNIKSHYCRTVESAKYDEIRSEWRVETKNTIE
GILEVYEAKFLVIATGENSEGYIPNVPGLNNFEGEVVHSKNYKSGSKYKTKEVLVVGCGN
SGMEIAYDLHNSGANPSIVVRSPFHVFNREIIHQGMRMVKYFSVGVVDTIITLWAKLKYG
DLSKYGIYRPKLGPFNLKNVTGKSAVIDVGTVEKIKEGSIKVVSSYITRIEKKKVVFENNME
KEFDAIVFATGYKSIANGWLKDYKYALNEKGMPKNAYPSHWKGDHGLYCAGLARRGLS
GVKIDAESIAEDINQTFNFHN*

(11) MtYUCCA11(Medtr6g086870)
MAHDHQKKDSMGKSRLVHGPIIVGAGPSGLAAAACLKQKGIPSQILERANCLASMWQLK
TYDRLRLHLPKQFCQLPLMPFPKGLPSYPTKQQFLSYLKAYADHFDINPIFGKQVVNAEF
DVVCGFWKIKTQEIMKNGDEIIIEYLCKWLIVASGENAEEVVPQIEGMEQFQGPILHTSLY
KSGSMFCGKNVLVVGCGNSGMEVCLDLCNHNAHPSLVVRDTVHILPQQIFGKSTFGLS
MWLLKWFSVQFVDKFLLLMSDLILGDTSQFGIERPKIGPLELKNLYGKTPVLDVGTVAQIK
TGKIKVCKGIKRLAHNAVEFVDGKVENFDAIILATGYRSNVPSWLKGSDMFSEKDGFPRK
PFPNGWKGEKGLYAVGFTKRGLLGSSIDAKRIAEDIETSWKSIKAKPLA*

(12) MtYUCCA12(Medtr7g099160)
MDMDLDEETILLYENVLKTARAKAVENPNDDDVDNLLDCAGALLTLHNVKDFTTSKEMLE
EAVTKFKKVTELNPDLHRPFWALGKTLTAQAILFSTRAEAKDHYELAYDYFLKAVEKNPK
NKLYRISLQEAAKKAPGRGPFTSAGFKQSAGTADPSSAGTENVPGTCPFSSAGTKRSIG
TAGLSSSAGTEDPSNLPDVDPLDGDAKVCESIDPPASRFISYLENYANKFEINPQFNECV
QSAKYDETSGLWRVKTNEVEYICRWLVVATGENAECVTPEIEGLSEFKGEVVYACDYKS
GKNFEGKKVLVVGCGNSGMELSLDLSNHHALPSMVVRSSVHVLPREIFGISTFELAVMM
LKWLPLWIVDKLLLILTWFILGDMEKYGIKRPSMGPLQLKNTVGKTPVLDIGALEKIRSGDI
NVVPGIKRINKNGEVELVNGEKLDIDAVVLATGYRSNVPSWLQEGEFFSKNGYPKMPFP
HGWKGNSGLYAVGFTKRGLSGASSDAVKIAQDIGKVWKQETKQKKQRTTACHRRCISQ

F*

(13) MtYUCCA13(Medtr7g099330)
MENLFRLADHQDFLSRRCIWVNGPVIIGAGPSGLATAACLREQGVPFVVLERADCIASLW
QKRTYDRLKLHLPKQFCQLPNLPMPEDFPEYPSKKQFISYLENYANKFEINPQFNECVQ
SAKYDETSGLWRVKTNEVEYICRWLVVATGENAECVTPEIEGLSEFKGEVVYACDYKSG
KNFEGKKVLVVGCGNSGMELSLDLSNHHALPSMVVRSSVHVLPREIFGISTFELAVMML
KWLPLWIVDKLLLILTWFILGDMEKYGIKRPSMGPLQLKNTVGKTPVLDIGALEKIRSGDIN
VVPGIKRINKNGEVELVNGEKLDIDAVVLATGYRSNVPSWLQEGEFFSKNGYPKMPFPH
GWKGNSGLYAVGFTKRGLSGASSDAVKIAQDIGKVWKQETKQKKQRTTACHRRCISQF*

(14) MtYUCCA14(Medtr8g432640)
MQEFTVVIVGAGPSGLAISACLTQNSISHIILEKEDCCASLWRKNAYDRLNLHLASEFCSL
PLMPHPPSGPTYLSKDQFLQYIDKYVEHFNIKPCYCRMVESAEYDEVGNKWRVQTKNT
QDDTLEVYVSNYLVIATGENSEGYIPNVPGLGNFEGEVMHSKYYKSGSKYESKNVLVVG
CGNSGMEIAYDLHNWGASASIVIRNPLHVFTREMIHQGMRMVKYLPVHIVDTYITLLAKL
KYGDLSKYGIYRPKEGPLHLKNITGKSAVIDVGTIGKIKEGAIKVVPSNITRIEKKKIVFENN
VEREFDAIVFATGYKSIANGWLKDYKYALNDKGMPKNAFPKHWKGDRGLYCGGLARRG
LWGVKVDAESIAQDINQNLNLHD*
