# Supplementary material for: Genome-Wide Identification, Expression Analysis, and Potential Roles under Abiotic Stress of the YUCCA Gene Family in Mungbean (Vigna radiata L.)
Source: Int J Mol Sci. 2023 Jan 13;24(2):1603. doi: 10.3390/ijms24021603 (PMC9866024; doi:10.3390/ijms24021603)
Supplement: Supplementary file 1 [file ijms-24-01603-s001.zip › Table S1 VrYUCCA Genes information.pdf]

Table S1. The information of *VrYUCCA* genes in mungbean

Name	Gene[a]	NCBI	Locus ID	Location	length (aa)	pI	M.W.	Predicted cell localizations
VrYUCCA2a	*Vradi01g04110*	XP_014508305.1	*LOC106767860*	Chr01 (6395222..6398559, complement)	425	9.03	47.57	Cytoplasm
VrYUCCA2b	*Vradi07g28320*	XP_014508261.1	*LOC106767822*	Chr07 (52005775..52008781)	416	9.00	46.39	Cytoplasm
VrYUCCA3	*Vradi08g03860*	XP_014511461.1	*LOC106770152*	Chr08 (6711112..6714058)	434	9.12	48.40	Cytoplasm
VrYUCCA4a	*Vradi05g09830*	XP_014500804.1	*LOC106761746*	Chr05 (18116316..18118574, complement)	408	9.54	45.61	Cytoplasm
VrYUCCA4b	*Vradi06g12650*	XP_014504238.1	*LOC106764471*	Chr06 (30511543..30514503, complement)	401	9.05	44.80	Cytoplasm
VrYUCCA6a	*Vradi05g09130*	XP_014500302.1	*LOC106761281*	Chr05 (17097676..17101207, complement)	427	7.96	48.04	Cytoplasm
VrYUCCA6b	*Vradi06g10150*	XP_014504106.1	*LOC106764337*	Chr06 (22339334..22343602, complement)	440	9.29	49.89	Chloroplast
VrYUCCA8a	*Vradi03g02780*	XP_014495524.1	*LOC106757377*	Chr03 (3873022..3875211)	423	8.79	47.52	Cytoplasm
VrYUCCA8b	*Vradi08g08480*	XP_014513031.1	*LOC106771549*	Chr08 (23774301..23776285)	423	8.85	47.73	Nucleus
VrYUCCA8c	*Vradi08g08490*	XP_014512724.1	*LOC106771284*	Chr08 (23808239..23810294)	424	8.85	47.86	Nucleus
VrYUCCA10	Not find	XP_014495430.1	*LOC106757305*	Chr03 (7146301..7149975, complement)	385	8.50	43.14	Cytoplasm
VrYUCCA11	Not find	XP_014522304.1	*LOC106778822*	NW_014541999.1 (311357..313142, complement)	399	8.76	44.64	Cytoplasm

Note: pI, isoelectric point; M.W. molecular weight. a, these Gene IDs were taken mung bean database (http://plantgenomics.snu.ac.kr/mediawiki-1.21.3/index.php/Main_Page) released in 2014 as the reference.
