# Supplementary material for: Genome-Wide Identification, Expression Analysis, and Potential Roles under Abiotic Stress of the YUCCA Gene Family in Mungbean (Vigna radiata L.)
Source: Int J Mol Sci. 2023 Jan 13;24(2):1603. doi: 10.3390/ijms24021603 (PMC9866024; doi:10.3390/ijms24021603)
Supplement: Supplementary file 1 [file ijms-24-01603-s001.zip › Table S2 Primers.pdf]

Table S2. All primers used in this study

Purpose	Name of primers	Sequences (5’-3’)
For RT-PCR and Real-time PCR	VrActin-3-F	CAGTGTCTGGATTGGAGGCT
	VrActin-3-R	GTCCTCGACCACTTGATG
	VrYUC2a-RTF	GGGAGTGACATGTTCAGTGAGAAAGATG
	VrYUC2a-RTR	TCATGCAAGTGGACAAGGAAACTCC
	VrYUC2b-RTF2	TGGTTGAAGGGGAGTGAGATGTTTTG
	VrYUC2b-RTR2	TTACGTGGTCTTAGCAGCTTTCCAGC
	VrYUC3-RTF2	CAAACGATGGAACTCCAAGAAACCC
	VrYUC3-RTR2	TTAGAAGTGTGATATGCACCTCCTGTGAC
	VrYUC4a-RTF2	ATGGAGTCTTGCAAGGGACCAGAG
	VrYUC4a-RTR2	GGAAATGGTGAGGGAAGGGCAT
	VrYUC4b-RTF1	GGAACACAGTGCATGTCCTTCCTAGG
	VrYUC4b-RTR1	CTCCTTCACACCTTCCATCACCTTTATG
	VrYUC6a-RTF	CAAGGCATCGTCTTCTTCAACGGT
	VrYUC6a-RTR	TGGGGAAGTGAGAAGGGAAGGG
	VrYUC6b-RTF	ATGCATTCTTGGATGGACTATTGCTTG
	VrYUC6b-RTR	CGCTCTAGGATTAGGCTTGGAACTCC
	VrYUC8a-RTF2	CTCAAAGAATGGATTCCCGAAGATG
	VrYUC8a-RTR2	TTAGAACTGAGAAATGCATCTTCTATGACATG

	VrYUC8b-RTF	TTCGCTTAGTTGATTGTGAGGAAACG
	VrYUC8b-RTR	AAGTGTGGTGGGAATGGAAGGTTG
	VrYUC8c-RTF2	ATGGAAAACTTATTTCGCCTAGTTAATTGTGA
	VrYUC8c-RTR2	AGATGGAGTTTTAATCTGTCGTAGGTTCGT
	VrYUC10-RTF2	GAGATCAACCAACAAGTGGCTCAAGG
	VrYUC10-RTR2	TCAGGGTTGTTGCATGAAGAAGCTTATAT
	VrYUC11-RTF	CAACAGTAATGGAATGCCTAAACCAGG
	VrYUC11-RTR	TTATTTCTGAATGAGTTTGAGGGAAGCTAAG
	AtActin2-F	TCCTTTGTTGCTGTTGACTACG
	AtActin2-R	ATTTTCTGTGAACGATTCCTGG
For prokaryotic expression	pMALVrYUC2a-F	TTCAGAATTCGGATCCATGGACTACTTGAAGGAACTAGAAGGAAAAAG
	pMALVrYUC2a-R	GCAGGTCGACTCTAGATCATGCAAGTGGACAAGGAAACTCC
	pMALVrYUC2b-F	TTCAGAATTCGGATCCATGGTTTTGATGGAGTACTTGAAGGAGG
	pMALVrYUC2b-R	GCAGGTCGACTCTAGATTACGTGGTCTTAGCAGCTTTCCAGC
	pMALVrYUC3-F	TTCAGAATTCGGATCCATGCATGACATTTGTCTCAACATGCC
	pMALVrYUC3-R	GCAGGTCGACTCTAGATTAGAAGTGTGATATGCACCTCCTGTGAC
	pMALVrYUC4a-F	TTCAGAATTCGGATCCATGGAGTCTTGCAAGGGACCAGAG
	pMALVrYUC4a-R	GCAGGTCGACTCTAGACTATGTACTCTTGAGCAAGATCATGTGGG
	pMALVrYUC4b-F	TTCAGAATTCGGATCCATGGGTTCTTGCAAACCCCAACA
	pMALVrYUC4b-R	GCAGGTCGACTCTAGATTATGAAGTGAGAAGGATGATATGTGAATTGC

pMALVrYUC6a-F	TTCAGAATTCGGATCCATGGACTATTGCATAAGAGAAATAGAGGGAA
pMALVrYUC6a-R	GCAGGTCGACTCTAGACTATGAATTTGGTTGCGGCAAGTG
pMALVrYUC6b-F	TTCAGAATTCGGATCCATGCATTCTTGGATGGACTATTGCTTG
pMALVrYUC6b-R	GCAGGTCGACTCTAGATCATGAATTTGATTGTGGAACATTAAATGAG
pMALVrYUC8a-F	TTCAGAATTCGGATCCATGGAGAACTTTTTTCGCCTAGTGGAC
pMALVrYUC8a-R	GCAGGTCGACTCTAGATTAGAACTGAGAAATGCATCTTCTATGACATG
pMALVrYUC8b-F	TTCAGAATTCGGATCCATGGAAAACTTTTTTCGCTTAGTTGATTG
pMALVrYUC8b-R	GCAGGTCGACTCTAGATCAGAACTGAGAAATGCAACGTCTATGAG
pMALVrYUC8c-F	TTCAGAATTCGGATCCATGGAAAACTTATTTCGCCTAGTTAATTGTGA
pMALVrYUC8c-R	GCAGGTCGACTCTAGATCAGAACTGAGAAATGCAACGTCTATGAG
pMALVrYUC10-F	TTCAGAATTCGGATCCATGCAGGAGGAAGATAAGGCCGAG
pMALVrYUC10-R	GCAGGTCGACTCTAGATCAGGGTTGTTGCATGAAGAAGCTTATAT
pMALVrYUC11-F	TTCAGAATTCGGATCCATGGAGATTGAAGTTCCTGTTGTGATTG
pMALVrYUC11-R	GCAGGTCGACTCTAGATTATTTCTGAATGAGTTTGAGGGAAGCTAAG
